# Enhanced stability and reusability of recombinant silicatein upon biomimetic metal–organic framework crystallization

**DOI:** 10.1039/d5sc05521k

**Published:** 2025-12-15

**Authors:** Tongtong Zhang, Xiangyu Wang, Jack D. Wright, George F. S. Whitehead, Jeremiah P. Tidey, Lu Shin Wong, Imogen A. Riddell

**Affiliations:** a Department of Chemistry, The University of Manchester Oxford Road Manchester M13 9PL UK imogen.riddell@manchester.ac.uk; b Manchester Institute of Biotechnology, University of Manchester 131 Princess Street Manchester M1 7DN UK L.s.Wong@manchester.ac.uk; c Department of Physics, University of Warwick Gibbet Hill Road Coventry CV4 7AL UK

## Abstract

Enzymes capable of performing selective chemical transformations under benign and environmentally friendly conditions are attractive alternatives to synthetic catalysts. The inherent instability of many enzymes is, however, an impediment to their widespread application. Here, we demonstrate that biomimetic crystallization provides a route to metal–organic framework (MOF)-enzyme composites that are stable in aqueous and organic solvents, and can be reused over multiple reaction cycles. We show that for silicatein, an enzyme with established stability challenges, this approach extends the enzymatic half-life at room temperature from one week to one month. We also demonstrate how changes in the MOF structure affect the solution processability, driving enhanced catalytic performance. Biomimetic MOF crystallization thus represents a robust approach to the stabilization of biocatalysts for process intensification.

## Abstract 2

Enzyme encapsulation within a 2D layered metal–organic framework stabilizes enzymes that are otherwise prone to aggregation and practical handling difficulties, whilst maintaining good reactant access to the enzyme.

## Graphical abstract

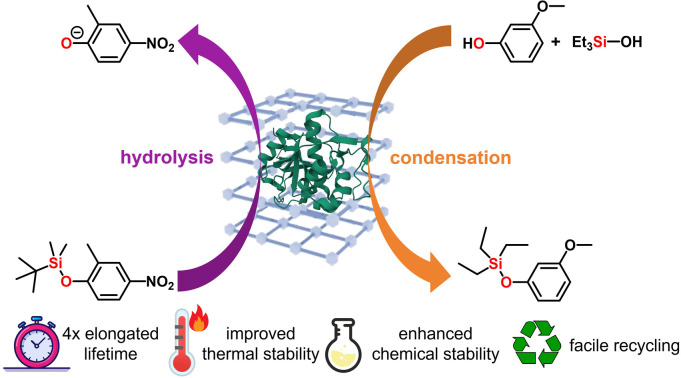

## Introduction

Enzymes are attractive alternatives^1–4^ to synthetic small-molecule catalysts which frequently employ organic solvents, elevated temperatures, and precious metals, and thus compromise industrial and governmental sustainability goals.^5,6^ Despite the benefits of enzyme-catalyzed transformations, many valuable enzymes are yet to be translated into industrial settings due to the high costs arising from lack of enzyme reusability and a requirement for specific storage and/or reaction conditions. Silicatein, a naturally derived enzyme capable of catalyzing Si–O bond hydrolyses and condensations, is an established biotechnological target. Specifically, silicatein facilitates the synthesis of organosiloxanes^7,8^ and crystalline metal oxides^9,10^ – materials with extensive applications in cosmetics, optics and catalysis.^11^ Application of silicatein in chemical synthesis is, however, yet to be realized due to its propensity to aggregate and precipitate from solution.^7,11^ To date, a variety of biochemical approaches have been investigated to improve the solubility of silcatein, with the most effective approach being the formation of a fusion protein of trigger factor (TF) and silicatein (TF-Silα).^7,12–14^ However, despite inclusion of the solubility tag, TF-Silα is still prone to oligomerization.^15^ Accordingly, the availability of a silicatein preparation that is robust and generically applicable remains unmet.

**Fig. 1:**
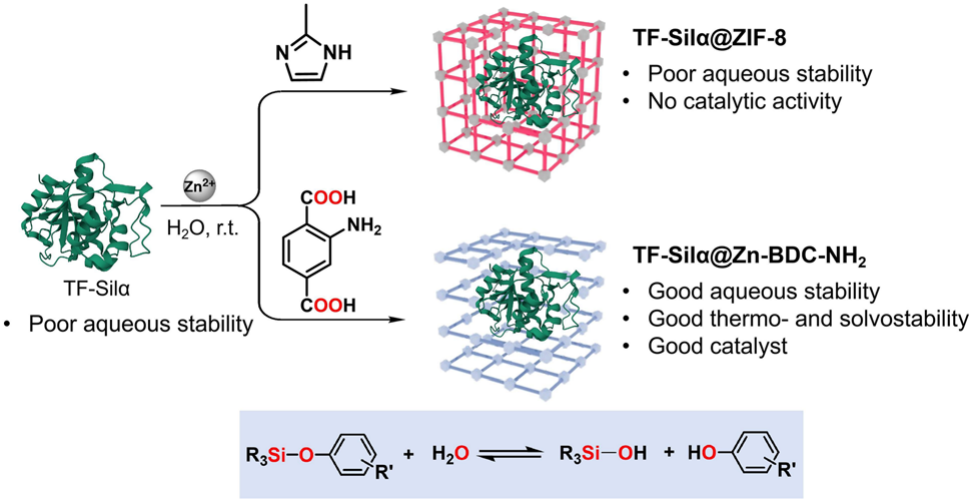
Schematic of biomimetic crystallization of TF-Silα in ZIF-8 (top, red) and Zn-BDC-NH_2_ (bottom, blue). Catalytic activity of encapsulated enzyme shown in blue box.

Biomimetic crystallization is emerging^16–18^ as a promising materials approach to protein stabilization, with reports detailing the enhanced thermal, chemical and biological stability of enzymes following encapsulation and the potential for enzyme reuse following multiple catalytic cycles.^19–21^ Here, we report the production and characterization of two TF-Silα@MOF composites derived from a zeolitic imidazole framework, ZIF-8, and a dicarboxylate-based MOF, Zn-BDC-NH_2_ (Fig. 1). These Zn-based MOFs were selected for enzyme immobilization due to their established biocompatibility and lack of toxicity,^22,23^ factors which minimize the risk of protein denaturation and will enable translation of these materials into sustainable chemical synthesis^24^ and therapeutic settings.^23,25^ Whilst TF-Silα@ZIF-8 shows negligible catalytic performance, TF-Silα@Zn-BDC-NH_2_ demonstrates comparable catalytic performance to the native TF-Silα, in addition to benefitting from enhanced enzyme stability, ease of recycling and reduced long-term storage requirements.

Here, we demonstrate that proteins with unmet solubility and stability challenges can be encapsulated within MOFs and the protective framework enables reuse of the enzyme as well as elongation of its half-life at room temperature by 4.4-fold.

## Results and discussion

### Biomimetic crystallization of TF-Silα

Initial experiments set out to evaluate MOF formation with TF-Silα in the presence of zinc(ii) nitrate and two separate organic linkers, 2-aminoterephthalic acid and 2-methylimidazole,^16,20,26^ with the aim of producing TF-Silα@Zn-BDC-NH_2_ and TF-Silα@ZIF-8, respectively. These ligands were chosen as they had been previously reported to be capable of generating framework materials with tunable properties including particle size, protein loading and hydrophobicity, each of which affects the encapsulated protein's catalytic performance.^27^ Following established protocols,^16,20,26^ we combined solutions of zinc(ii) nitrate, the appropriate organic linker and freshly isolated TF-Silα (SI Section S3). The isolated precipitates were washed with water and methanol and then analyzed to determine their crystal structure, particle size, morphology, and to confirm the presence of Silα.

### Enzyme encapsulation in Zn-BDC-NH_2_

Firstly, the encapsulation efficiency (EE, percentage of encapsulated protein) and loading content (LC, weight-percentage of protein in MOF) were calculated by quantifying the residual protein in the supernatant. In TF-Silα@Zn-BDC-NH_2_ high EE values were achieved (96%), with LC values of approximately 10% (Table S3), which compares favorably with EE and LC values reported for other enzyme@MOF composites reported in the literature.^16,19,20^

The Zn-BDC-NH_2_ biocomposite was then characterized using vibrational spectroscopy (FTIR), thermogravimetric analysis (TGA) and energy dispersive X-ray (EDX) spectroscopy. The FTIR spectrum for TF-Silα@Zn-BDC-NH_2_ included a peak between 1600 and 1690 cm^−1^(Fig. S14a) that would be consistent with the amide I band from the protein.^28^ However, the carboxylate stretch in the parent Zn-BDC-NH_2_ MOF also appeared in this region of the spectrum and thus could not confirm the incorporation of TF-Silα. Closer inspection of the second-derivative FTIR spectrum (Fig. S15a and c) revealed that TF-Silα@Zn-BDC-NH_2_ retained peaks attributed to α-helical peptide sequences (1659, 1652, and 1645 cm^−1^),^29^ these peaks were absent in the pristine MOF providing further evidence of protein encapsulation (Fig. S15a and c). As evidenced by the second-derivative FTIR spectrum, the characteristic secondary structures of TF-Silα including α-helice and β-sheets were preserved,^29^ indicating the protein retained its conformation upon immobilization. This result was supported by CD spectroscopy experiments (Fig. S16a) which evaluated potential changes in the protein structure during the MOF nucleation process.^30^ CD spectra recorded for TF-Silα in the presence and absence of the BDC-NH_2_ ligand were comparable (Fig. S16a), with both spectra clearly displaying α-helical features.

TGA of TF-Silα@Zn-BDC-NH_2_ revealed a three-step weight decrease (Fig. S17). By comparison to previous reports of protein@MOF TGAs,^31,32^ the initial mass loss at lower temperatures (*T* < 200 °C) was attributed to water evaporation from the framework, followed by loss of the protein between 200 and 400 °C, with mass loss above 400 °C being attributed to MOF decomposition.^33^ For TF-Silα@Zn-BDC-NH_2_ a 20% weight loss was found from 300 to 400 °C, whilst only 12% mass reduction was observed for the parent Zn-BDC-NH_2_ in the absence of protein, which was attributed to BDC-NH_2_ linker decomposition (SI Fig. S17c and d). Thus, this difference in the mass loss (*i.e.* 8%) could be assigned to the loss of TF-Silα. However, as the protein loading and residual solvent content in the biocomposite vary amongst batches (Table S3), TGA only offers an estimation of the enzyme@MOF composition. Additionally, the EDX spectra for the TF-Silα@Zn-BDC-NH_2_ particles clearly indicated signals corresponding to sulfur (Fig. S18), which must originate from cysteine residues within the TF-Silα.

**Fig. 2:**
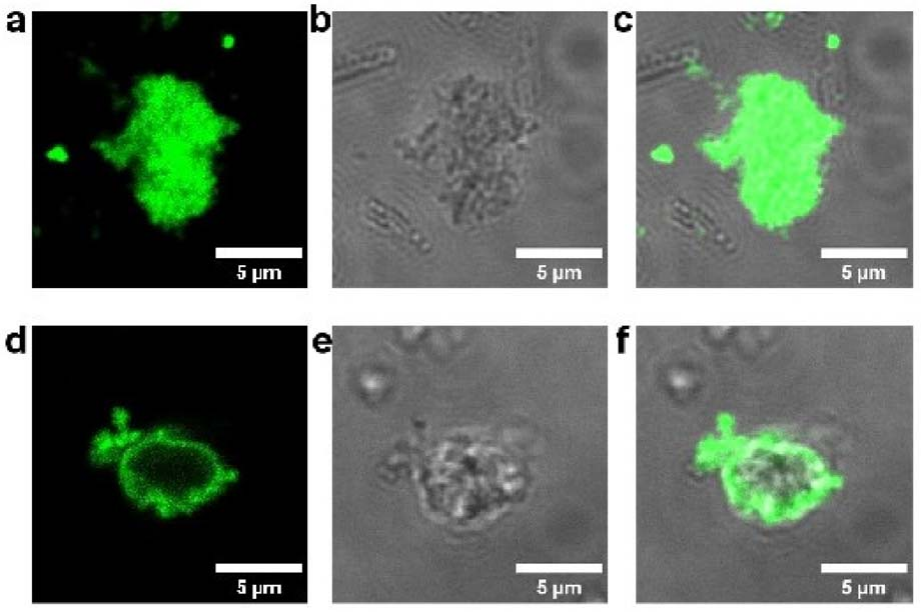
Confocal laser scanning micrograph of TF-Silα-eGFP@Zn-BDC-NH_2_; (a) dark field excited at 488 nm, (b) bright field, and (c) merged. Confocal laser scanning micrograph of TF-Silα-eGFP on Zn-BDC-NH_2_: (d) dark field excited at 488 nm, (e) bright field and (f) merged.

To determine protein localization within the MOF particles, a fusion protein comprised of TF-Silα and enhanced green fluorescence protein (TF-Silα-eGFP) was produced and encapsulated within the MOF. The TF-Silα-eGFP@Zn-BDC-NH_2_ was assembled using analogous procedures to those previously employed, and no significant differences in the PXRD patterns (Fig. S31b) were recorded (see discussion below; Fig. S31) indicating no changes to the MOF structure following introduction of the eGFP domain. The biocomposite was then imaged by confocal laser scanning microscopy (CLSM) and co-localization of the MOF particles and green fluorescence was observed (Fig. 2). Subsequent optical sectioning of the MOF particles showed that the fluorescence corresponding to the eGFP domain was present uniformly throughout all sections (Fig. S20 and S23), which is consistent with protein impregnation within the MOF structure. In contrast, control experiments in which TF-Silα-eGFP was mixed with preformed Zn-BDC-NH_2_ particles resulted in the formation of aggregates with non-fluorescent cores indicative of protein binding exclusively on the surface of the MOF (Fig. 2, S24).

### Topology and morphology of TF-Silα@Zn-BDC-NH_2_

**Fig. 3:**
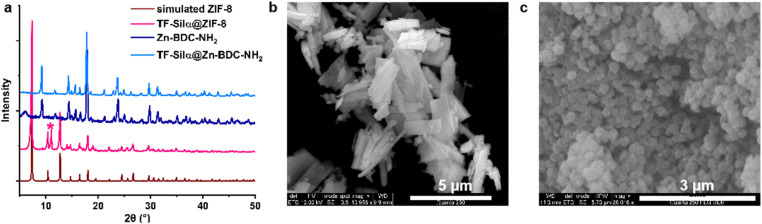
Topology and morphology study. (a) PXRD patterns of simulated ZIF-8 (from ref. 35), TF-Silα@ZIF-8, synthesized Zn-BDC-NH_2_ and TF-Silα@Zn-BDC-NH_2_. The PXRD pattern for TF-Silα@ZIF-8 is consistent with primarily a sodalite structure with small amount (21.5%) of ZIF-CO_3_-1 mixture (marked with asterisk*). The PXRD patterns for Zn-BDC-NH_2_, both in the presence and absence of TF-Silα, correspond with a known 2D layered MOF structure (ref. 26). SEM images of (b) TF-Silα@Zn-BDC-NH_2_ plates and (c) three-dimensional TF-Silα@ZIF-8 crystals.

The materials precipitated following biomimetic crystallization were also characterized using single-crystal electron diffraction (3D ED), PXRD, DLS and SEM. Three single crystals of TF-Silα@Zn-BDC-NH_2_ were selected under cryogenic conditions for electron diffraction analysis (SI S4.5). Solution and refinement of the data supported formation of a previously reported 2D layered MOF structure.^26^ In this MOF, each zinc(ii) ion is bound to three BDC-NH_2_ linkers and two water molecules, with the water protons directed out into the interlayer space (Fig. S25). The PXRD pattern (Fig. 3a) of the bulk material was consistent with the single-crystal data and no features corresponding to the protein were observed in either the 3D ED or PXRD data sets, which is consistent with a disordered arrangement of TF-Silα within the crystalline MOF. Moreover, the TF-Silα@Zn-BDC-NH_2_ diffraction patterns appeared identical to those of the parent structure lacking protein, Zn-BDC-NH_2_, implying that enzyme incorporation did not influence the long-range order of this MOF (Table S6).

The crystal size and shape distributions were also studied by DLS and SEM. DLS data showed two distinct populations of TF-Silα@Zn-BDC-NH_2_ crystals centered at hydrodynamic diameters (*D*_h_) of 172 nm and 1.0 µm (Fig. S33). SEM images of TF-Silα@Zn-BDC-NH_2_ (Fig. 3b) displayed plate-shaped crystals with average diameters of around 1–3 µm, alongside some smaller nanoparticles, consistent with the DLS results.

### Synthesis and characterisation of TF-Silα@ZIF-8

As a comparison, TF-Silα@ZIF-8 was also prepared since ZIF-8 is one of the most studied MOFs for biomimetic crystallization. This biocomposite also shows relatively high EE (86%) and LC (14%) values compared to reported enzyme@MOF systems.^16,19,20^ In FTIR spectra, an amide I band^28^ was found at 1670 cm^−1^ which is absent in the spectrum of the enzyme-free ZIF, indicating TF-Silα incorporation. Second derivative analysis of the FTIR spectrum indicates a redshift (4–7 cm^−1^) of α-helical peaks, consistent with protein-MOF interactions,^34^ but overall the peaks corresponding to secondary structure are preserved, supporting retention of the protein structure upon ZIF-8 encapsulation (Fig. S15b and d). This result is consistent with CD experiments which indicate retention of α-helical features during mixing of TF-Silα with 2-methylimidazole, as occurs during MOF nucleation^30^ (Fig. S16b). TGA suggests no significant mass loss for pure ZIF-8 between 200 and 350 °C. In contrast, a 9.5% weight loss was observed across this temperature range for TF-Silα@ZIF-8, corresponding to protein degradation. The EDX spectrum of the MOF particles also exhibited a sulfur signal that further confirmed enzyme incorporation. As with the TF-Silα@Zn-BDC-NH_2_ composite, confocal micrographs of TF-Silα-eGFP@ZIF-8 indicated uniform fluorescence throughout all optical sections (Fig. S19 and S20), supporting the protein encapsulation within the MOF rather than adsorption on to its surface.

The topology and morphology of TF-Silα@ZIF-8 were also investigated. The PXRD pattern showed a primary topology of sodalite^35^ (78.5%) with the peak at 11 degrees 2*θ* (Fig. S27 and S28) indicating a minor component of ZIF-CO_3_-1 (21.5%) within the mixture.^36^ In contrast, the attempted synthesis of ZIF-8 in the absence of TF-Silα, but under otherwise identical conditions, occurred over a longer timeframe, gave lower isolated yields, and the PXRD patterns indicated the exclusive formation of ZIF-L (ref. 37 and 38) (Fig. S26). SEM revealed leaf-like particles of this material which also corresponds to the characteristic morphology of ZIF-L^37^ (Fig. S36). This result is consistent with previous reports where the ZIF-8 sodalite topology is favored over other crystalline forms when proteins that promote biomimetic crystallization are present.^39^ The particle size distribution was further investigated with DLS. In contrast to TF-Silα@Zn-BDC-NH_2_, TF-Silα@ZIF-8 exhibited a large size polydispersity, with hydrodynamic diameters (*D*_h_) ranging from 200 nm to 20 µm (Fig. S33). SEM images of TF-Silα@ZIF-8 revealed three-dimensional particles, similar to the rhombic dodecahedron morphology as previously reported,^40^ and particle sizes were observed around 200 nm (Fig. 3c and S35). This difference in the apparent sizes from the two analyses was attributed to particle aggregation due to the hydrophobic nature of ZIF-8.^27^

### Catalytic performance of encapsulated TF-Silα

**Fig. 4:**
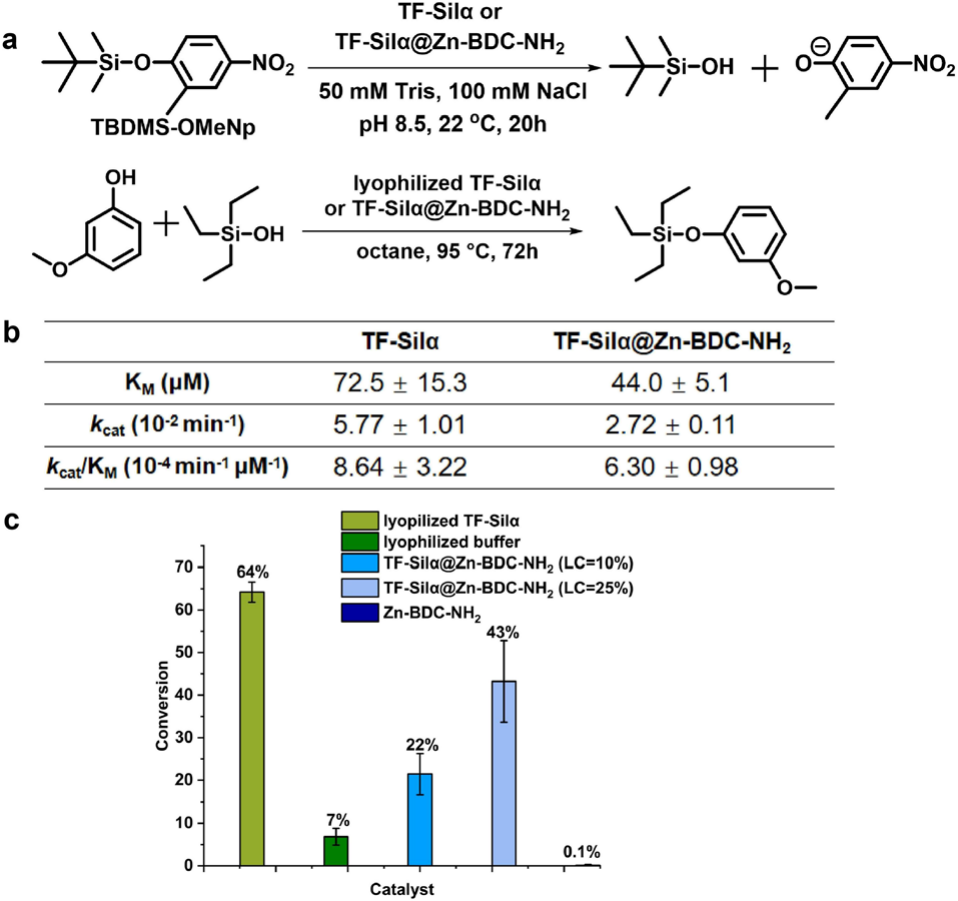
Catalytic performance of free and encapsulated TF-Silα. (a) Schemes representing the hydrolysis (top) and condensation reactions (bottom). (b) Apparent kinetic parameters of free (ref. 30) and encapsulated TF-Silα catalyzing the hydrolysis of TBDMS-OMeNp. Results are averaged from 3 measurements (c) Percentage conversions of 3-methoxyphenol to triethyl(3-methoxyphenoxy)silane. The reaction was catalyzed by lyophilized or encapsulated TF-Silα (loading content 10% or 25%) and their negative controls (lyophilized buffer and pure MOFs). The total TF-Silα concentration in the lyophilised and encapsulated TF-Silα preparations was kept constant at 67 µM in the reaction mixtures.

To quantify the enzymatic activity of the immobilized TF-Silα with regard to Si–O bond hydrolysis, a colorimetric assay involving the hydrolysis of the chromogenic substrate tert-butyldimethyl(2-methyl-4-nitrophenoxy)silane (TBDMS-OMeNp)^41^ was performed (Fig. 4a). Here, hydrolysis of the scissile bond results in the release of a highly absorbing nitrophenolate that can be quantified by UV-Vis spectrophotometry. In these assays, the TF-Silα@Zn-BDC-NH_2_ particles maintained an apparently stable colloid for over 20 hours (Fig. S38) and the hydrolytic activity could be measured with a high degree of reproducibility (Fig. 4b and S40). In contrast, in analogous experiments the TF-Silα@ZIF-8 particles precipitated rapidly resulting in large measurement errors (Fig. S38). The recorded hydrolytic activity was, however, consistent with background hydrolysis from the MOF and did not support any enzymatic activity (Fig. S39).

Since the overall reaction rate is dependent on both substrate diffusion within the matrix and the enzyme activity, the effectiveness factor was calculated to identify any differences between the mass transfer in TF-Silα@Zn-BDC-NH_2_ and the free enzyme.^42,43^ It was found that there was no significant change in effectiveness factor (Table S7) when increasing the substrate concentration from 20 µM to 60 µM, suggesting that mass transfer limitations are insignificant. The Michaelis–Menten model of enzyme kinetics could therefore be applied to these enzyme@MOF systems.^44,45^ The apparent Michaelis–Menten kinetic parameters *K*_M_, *k*_cat_ and *k*_cat_/*K*_M_ were determined and compared with parameters obtained for the free enzyme^41^ (Fig. 4b). It was found that the apparent *k*_cat_ for TF-Silα@Zn-BDC-NH_2_ was similar but slightly slower than for the free TF-Silα. The apparent substrate binding was, however, improved upon encapsulation as evidenced by lower *K*_M_ compared to free TF-Silα. The reduced *K*_M_ value might indicate reduced conformational freedom of the enzyme upon encapsulation that enhances enzyme–substrate affinity.^33,46^

Overall, TF-Silα@Zn-BDC-NH_2_ displays comparable hydrolysis activity to free TF-Silα as indicated by the similarity in the catalytic efficiency (*k*_cat_/*K*_M_) values. The observation that TF-Silα@Zn-BDC-NH_2_ outperforms TF-Silα@ZIF-8 in catalysis is attributed to the 2D layered structure of the former, which facilitates substrate diffusion between the interlayer spaces and renders the encapsulated enzyme more accessible. This is consistent with previous reports which highlight substrate diffusion as a key challenge for three-dimensional MOF structures.^47,48^

Next, the condensation activity of the immobilized enzyme was compared with the lyophilized TF-Silα.^49^ Here, a model reaction involving 3-methoxyphenol and triethylsilanol as substrates was investigated (Fig. 4a). The condensation product was quantified by GC-MS following 72 hours heating at 95 °C in octane. The thermal and chemical robustness of the TF-Silα@MOFs enabled their direct use in the reaction. Both lyophilized and encapsulated enzyme gave higher conversions compared to the negative control groups consisting of either the additives used to prepare the lyophilized enzyme (phosphate buffer salts and 18-crown-6) or the MOF alone without enzyme (Fig. 4c). It was found that increasing protein loading (LC) from 10% to 25%, increased the reaction conversion by two-fold (22 to 43%) even at equivalent quantities of enzyme.

As a comparison, TF-Silα@ZIF-8 at 10% LC showed 26% conversion, similar to TF-Silα@Zn-BDC-NH_2_. However, control studies with ZIF-8 alone gave a 6% conversion, attributed to general base catalysis by its 2-methyl imidazole linkers (Fig. S42). Overall, TF-Silα@Zn-BDC-NH_2_ retained both hydrolysis and condensation activity and when the protein loading in the MOF was properly tuned TF-Silα@Zn-BDC-NH_2_ retained ∼70% of the activity observed for TF-Silα in both reactions.

### Stability and reusability of TF-Silα@Zn-BDC-NH_2_

As encapsulation has been shown to improve protein stability under conditions that are not ordinarily biocompatible^16,27,46^TF-Silα@Zn-BDC-NH_2_ was next evaluated to see if this material exhibited enhanced thermal and chemical stability. Here, the TF-Silα free enzyme and TF-Silα@Zn-BDC-NH_2_ were both exposed to elevated temperatures (50 °C and 80 °C) or the organic solvents dioxane, methanol and THF (as representative examples of non-polar and polar solvents) for 2 hours prior to measuring their hydrolytic activity (see S5.6). PXRD analysis following heat or solvent treatment confirmed no structural degradation of the MOF had taken place under any of the conditions.

**Fig. 5:**
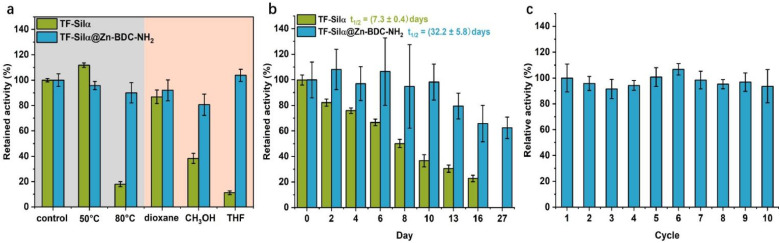
Stability and reusability investigation. (a) Relative activity of TF-Silα and TF-Silα@Zn-BDC-NH_2_ after exposure to elevated temperature or organic solvents for 2 h. (b) Retained activity of TF-Silα and TF-Silα@Zn-BDC-NH_2_ as a function of time when stored in buffered solution at room temperature. The half-life of free and encapsulated enzyme at 20 °C is found to be 7.3 ± 0.4 days and 32.2 ± 5.8 days, respectively. The activity was calculated as the initial rate during first 100 minutes, and the non-treated control group was normalized to 100% activity. (c) Relative activity of recycled TF-Silα@Zn-BDC-NH_2_ over ten cycles; results are averaged from three measurements. Error bars represent standard error of mean.

In terms of thermotolerance, little difference in the hydrolytic activity between the encapsulated and free enzyme was observed at 50 °C. Enhanced stability was, however, clearly demonstrated for the encapsulated proteins that had been heat-treated at 80 °C for 2 h, whereby the free silicatein lost 80% of its hydrolytic activity relative to the untreated TF-Silα control whilst the encapsulated enzyme exhibited only a minor reduction in activity (∼10%, Fig. 5a).

Similarly, enhanced chemical stability was observed for TF-Silα@Zn-BDC-NH_2_ compared with the free enzyme, especially when treated with the polar solvents methanol and THF (Fig. 5a). Polar solvents are well documented to disrupt the secondary and tertiary structures of proteins resulting in loss of enzymatic activity.^49–51^ Here, confinement inside the MOF restricts protein unfolding, thus inhibiting loss of catalytic activity.

To investigate the longevity of biocatalytic activity, TF-Silα and TF-Silα@Zn-BDC-NH_2_ were stored at room temperature for 16–27 days and their hydrolytic activity was monitored over time. Whilst a gradual decrease in hydrolytic activity was observed for the free enzyme over time, no significant loss of activity for the TF-Silα@Zn-BDC-NH_2_ was observed in the first 10 days followed subsequently by a gradual decline (Fig. 5b). Even so, the encapsulated enzyme retained ∼70% of its original activity after 27 days, while the free enzyme showed no activity after this time. The half-lives (*t*_1/2_) of TF-Silα and TF-Silα@Zn-BDC-NH_2_ at 20 °C were determined to be to be (7.3 ± 0.4) days and (32.2 ± 5.8) days, respectively. This result suggests encapsulation elongates the TF-Silα lifetime 4.4-fold and demonstrates the enhanced stability of TF-Silα@Zn-BDC-NH_2_ over the native protein.

In regard to the ease of recycling of the immobilized enzyme, the encapsulated enzyme particles were found to be readily separated by centrifugation post-reaction. These recovered enzyme@MOF particles were then subjected to nine further reaction cycles without any significant loss of activity (Fig. 5c). Since the free TF-Silα cannot be separated from the reaction mixture, a comparative experiment was carried out whereby a solution of TF-Silα was left to stand at 22 °C for different time periods. These time periods were chosen to correspond to the amount of time the enzyme would have been exposed to the enzyme@MOF reaction conditions (0–100 h) prior to hydrolysis, mimicking the reaction conditions during the catalyst recycling process. In this case, only 50–60% of the activity was retained relative to freshly defrosted isolated enzyme (Fig. S43). The ease of separation and the enhanced stability of the TF-Silα@Zn-BDC-NH_2_ both, therefore, contribute to its recyclability, enabling TF-Silα@Zn-BDC-NH_2_ to be reused under standard assay conditions for over 10 cycles without any obvious reduction of bioactivity. In contrast, in absence of the MOF each aliquot of enzyme must be freshly defrosted prior to use.

## Conclusions

Encapsulation of the otherwise unstable protein TF-Silα through biomimetic crystallization within the 2D layered MOF, Zn-BDC-NH_2_, is shown to significantly improve its operational stability. Specifically in the case of TF-Silα@Zn-BDC-NH_2_, biocatalytic activity is retained in both the model silyl ether hydrolysis and formation reactions, showing that neither the self-assembly process nor the framework deactivates the protein. Furthermore, the encapsulated TF-Silα shows enhanced thermo- and solvostability in comparison to the free enzyme. Additionally, excellent reusability was demonstrated over ten catalytic cycles with the half-life of TF-Silα at room temperature extended 4.4-fold upon MOF immobilization. This increase in room temperature stability presents opportunities to improve the stability and practical handling of fragile proteins, circumventing the need for cold storage for periods as long as a week. In contrast, despite being the most extensively studied biomimetic crystallization material, the TF-Silα@ZIF-8 biocomposite suffered from poor colloidal stability which negatively impacted its catalytic performance.

Overall, biomimetic crystallization offers a promising approach to stabilize fragile enzymes for process intensification. Future work will look to diversify the range of MOFs suitable for encapsulation enabling application of this technology to other sectors including the development of non-toxic MOFs for use in delivery of therapeutic proteins.

## Author contributions

Conceptualization, T. Z., L. S. W. and I. A. R; methodology, T. Z.; Investigation, T. Z., X. W., J. D. W., G. F. S. W., J. P. T.; formal analysis, T. Z.; validation, T. Z., L. S. W. and I. A. R; writing – original draft, T. Z.; writing – review & editing, T. Z., L. S. W. and I. A. R.; visualization, T. Z., I. A. R; resources, supervision and funding acquisition, L. S. W. and I. A. R.

## Conflicts of interest

There are no conflicts to declare.

## Supplementary Material

SC-OLF-D5SC05521K-s001

SC-OLF-D5SC05521K-s002

## Data Availability

The data supporting this article have been included as part of the supplementary information (SI). Supplementary information: substrate and calibrant synthesis, enzyme and enzyme@MOFs preparation, FTIR, CD, TGA, EDX, CLSM, 3D ED, PXRD, DLS, SEM and enzymatic activity study is available. See DOI: https://doi.org/10.1039/d5sc05521k. Supplementary crystallographic data in the form of the CIF is available at CCDC 2418698: Experimental Crystal Structure Determination, 2025, DOI: 10.5517/ccdc.csd.cc2m5vj6.^52^
