# Supplementary material for: Enhanced stability and reusability of recombinant silicatein upon biomimetic metal–organic framework crystallization
Source: Chem Sci. 2025 Dec 15;17(6):3141–7. doi: 10.1039/d5sc05521k (PMC12705213; doi:10.1039/d5sc05521k)
Supplement: SC-017-D5SC05521K-s001 [file SC-017-D5SC05521K-s001.pdf]

Supplementary Information (SI) for Chemical Science.
This journal is © The Royal Society of Chemistry 2025

Supporting information for:

Enhanced stability and reusability of recombinant silicatein upon biomimetic metal-organic framework crystallization

Tongtong Zhang,[1,2] Xiangyu Wang,[1] Jack D. Wright,[1] George F. S. Whitehead,[1] Jeremiah P. Tidey,[3] Lu Shin Wong[2]* and Imogen A. Riddell[1]*

1. Department of Chemistry, University of Manchester, Oxford Road, Manchester, M13 9PL, United Kingdom
2. Manchester Institute of Biotechnology, University of Manchester, Oxford Road, Manchester, M13 9PL, United Kingdom
3. Department of Physics, University of Warwick, Gibbet Hill Road, Coventry, CV4 7AL, UK

Table of Contents

S1. Synthesis of enzyme substrates and GC calibrants 3
S1.1 Materials 3
S1.2 Synthesis of tert-butyldimethyl(2-methyl-4-nitrophenoxy) silane (TBDMS-OMeNp)3
S1.3 Synthesis of triethyl(phenoxy)silane 5
S1.4 Synthesis of triethyl(m-methoxyphenoxy)silane 7
S1.5 Synthesis of triethyl(p-methoxyphenoxy)silane 9
S2. Expression, purification and lyophilization of recombinant silicatein 11
S3. Biomimetic crystallisation of TF-Silα 12
S3.1 Synthetic procedure for preparation of TF-Silα@Zn-BDC-NH_2 12
S3.2 Synthetic procedure for preparation of Zn-BDC-NH_2 13
S3.3 Synthetic procedure for preparation of TF-Silα-eGFP@Zn-BDC-NH_2 13
S3.4 Synthetic procedure for preparation of TF-Silα-eGFP on MOFs 13
S3.5 Synthetic procedure for preparation of TF-Silα@ZIF-8 13

S3.6 Synthetic procedure for preparation of ZIF-8 13
S3.7 Synthetic procedure for preparation of TF-Silα-eGFP@ZIF-8.................................14
S3.8 Protein quantification 14
S4. Characterization of TF-Silα@MOF composites 15
S4.1 Fourier transform infrared spectroscopy (FTIR) analysis 15
S4.2 Circular Dichroism (CD) 17
S4.3 Thermogravimetric analysis (TGA) 18
S4.4 Protein Localisation via Energy Dispersive Spectroscopy (EDX) and Confocal Laser Scanning Microscopy (CLSM) 19
S4.5 Electron diffraction (ED)...22
S4.6 Powder X-ray diffraction (PXRD) 25
S4.7 Particle size analysis via Dynamic light scattering (DLS) and scanning electron microscopy (SEM) 30
S5. Activity study of free and encapsulated TF-Silα 31
S5.1 Hydrolytic activity of TF-Silα...31
S5.2 Hydrolytic activity of TF-Silα@MOF composites 32
S5.3. Effectiveness factor of encapsulated TF-Silα...34
S5.4 Condensation activity of TF-Silα and TF-Silα@MOF composites 35
S5.5. GC-MS calibration 35
S5.6 Comparison of stability of free and biomineralised TF-Silα 37
S5.7 Determination of half-life for free and encapsulated TF-Silα.................................38
S5.8 Reusability test of free and biomineralised TF-Silα..38
S5.9 Optimization of condensation reaction..39
S6. References:...40

S1. Synthesis of enzyme substrates and GC calibrants

S1.1 Materials

All reagents were purchased from Sigma-Aldrich or Fluorochem and used without further purification. Pierce™ detergent compatible Bradford assay kit was purchased from Thermo Fisher scientific, containing a commercial Coomassie dye solution. The TF-Silα-Strep enzyme was heterologously produced in *E. coli*, isolated and formulated according to previously reported procedures.[1] Aliquots of the isolated protein solutions were flash frozen in liquid nitrogen and stored at -80°C until needed. For the subsequent experiments the enzyme solutions were defrosted by standing at room temperature for no longer than 1 hour before the start of the experiments.

Cell lysis was performed using a Bandelin Sonoplus HD2070 probe sonicator. The lysate was purified with a 5 mL Strep-Tactin® Superflow® high capacity column. UV-vis absorbances for the hydrolytic assays were measured in 96-well microtiter plates using Tecan Spark® Multimode Microplate Reader and Biotek Synergy HT Multi-Mode Microplate Reader. The execution and analysis of the silyl condensation reactions were carried out using the same procedures as previously reported.[2, 3] The results were analysed with the aid of authentic samples of the product silyl ether (see below).

S1.2 Synthesis of tert-butyldimethyl(2-methyl-4-nitrophenoxy) silane (TBDMS-OMeNp)

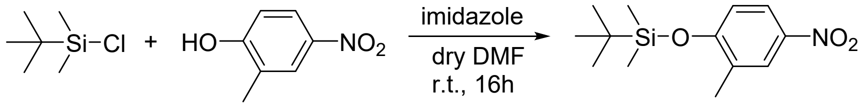

Tert-butyldimethyl(2-methyl-4-nitrophenoxy)silane (TBDMS-OMeNp) was prepared according to previously described procedures.[3] To a solution of 2-methyl-4-nitrophenol (853.6 mg, 5.57 mmol) and imidazole (690.5 mg, 10.14 mmol) in dry DMF (5 mL) was added tert-butyldimethylsilyl chloride (819.0 mg, 5.43 mmol) under an inert atmosphere. The mixture was stirred at room temperature for 16 hours after which H_2O (25 mL) was added. The mixture was then extracted three times with ethyl acetate (30 mL x 3), the organic extracts were combined and dried over anhydrous Na_2SO_4 before being concentrated under vacuum. The residue was purified by column chromatography (hexane:EtOAc, 7:3) to yield the final product as a pale yellow solid (962 mg, 66%). ^{1}H NMR (500 MHz, $CDCl_3$, 298 K) δ 8.06 (d, *J* = 2.8 Hz, 1H, ArH), 7.99 (dd, *J* = 8.9, 2.9 Hz, 1H, ArH), 6.80 (d, *J* = 8.9 Hz, 1H, ArH), 2.27 (s, 3H, $ArCH_3$), 1.03 (s, 9H, $C(CH_3)_3$), 0.28 (s, 6H, $SiCH_3$). ^{13}C NMR (126 MHz, $CDCl_3$, 298 K) δ 160.1, 141.6, 130.3, 126.7, 123.3, 118.0, 25.7, 18.4, 17.1, -4.0. MS (ESI): m/z calculated for $C_{13}H_{22}O_3NSi$ ($[M+H]^+$) 268.1363, found 268.1372. Data is consistent with previous report.[3]

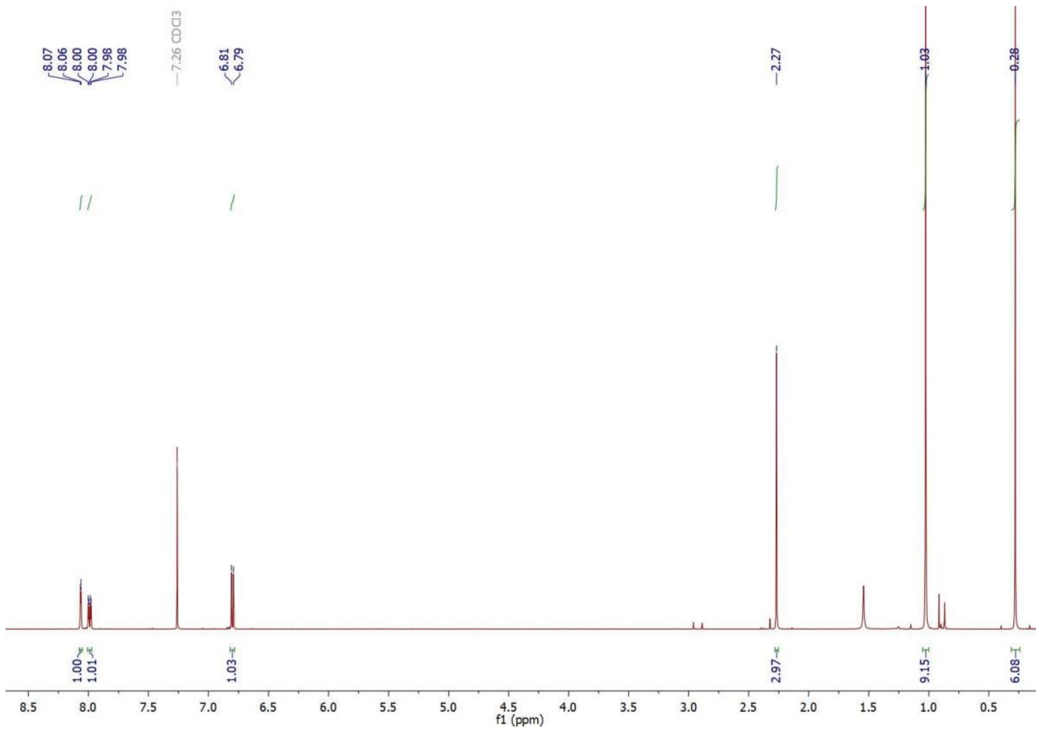

Figure S1. ^{1}H NMR (500 MHz, $CDCl_3$, 298 K) spectrum of TBDMS-OMeNp

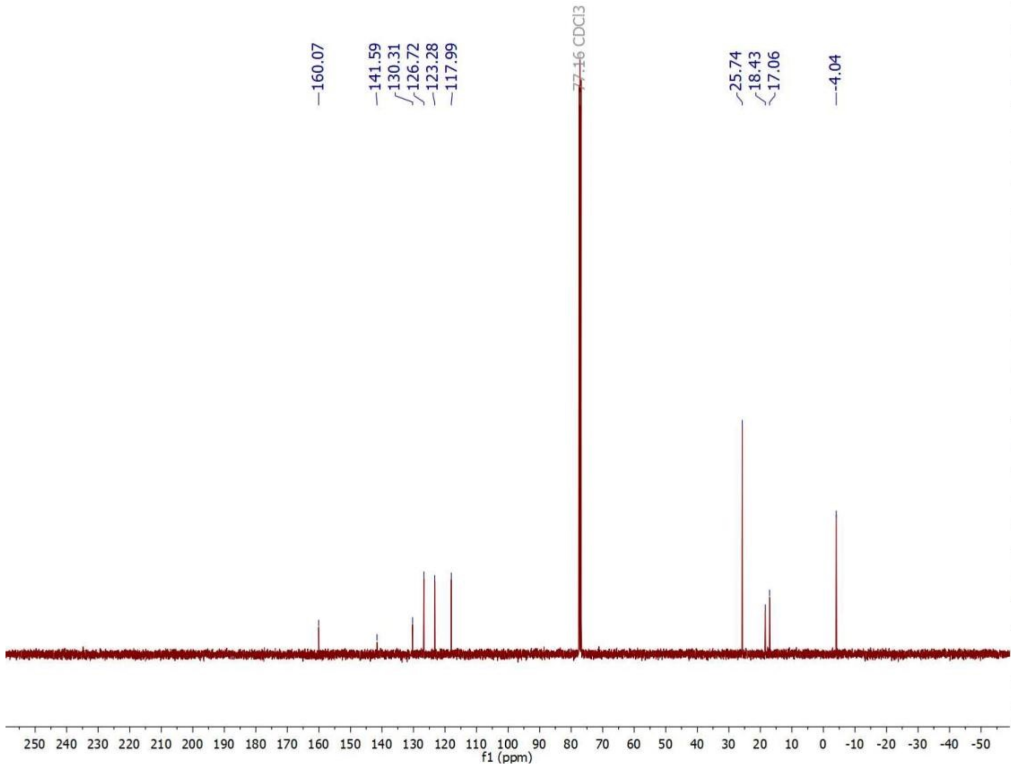

Figure S2. ^{13}C NMR (126 MHz, $CDCl_3$, 298 K) spectrum of TBDMS-OMeNp

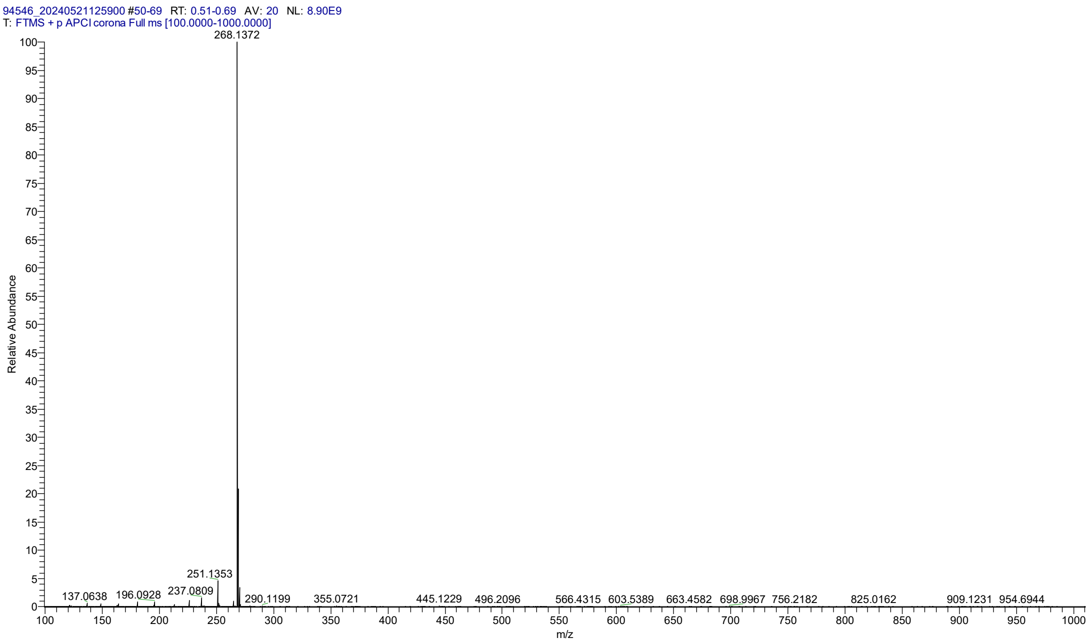

Figure S3. Mass spectrum (ESI+) for TBDMS-OMeNp: 268.1372 $[M+H]^+$.

S1.3 Synthesis of triethyl(phenoxy)silane

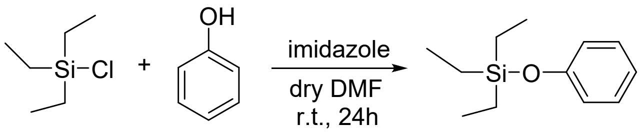

Triethyl(phenoxy)silane was prepared according to previously described procedures[2]. Chlorotriethylsilane (1.2 mL, 7.1 mmol) was added dropwise to a solution of phenol (562 mg, 6.0 mmol) and imidazole (1003 mg, 14.7 mmol) in dry DMF (6 mL) under an inert atmosphere. The solution was stirred at room temperature for 24 hours, diethyl ether (150 mL) added and the mixture washing twice with water (60 mL x 2). The organic phase was dried over anhydrous Na_2SO_4 and concentrated under vacuum. The residue was then purifed by silica gel chromatography (EtOAc:hexane, 1:30) to yield the desired product as a colourless oil (904 mg, 72%). ^{1}H NMR (500 MHz, $CDCl_3$, 298 K) δ 7.21-7.26 (m, J = 7.8 Hz, 2H, ArH), 6.95 (t, J = 7.3 Hz, 1H, ArH), 6.85-6.87 (d, J = 7.8 Hz, 2H, ArH), 1.01 (t, J = 7.9 Hz, 9H, CH_3), 0.73-0.78 (q, J = 7.9 Hz, 6H, CH_2).^{13}C NMR (126 MHz, $CDCl_3$, 298 K) δ 155.7, 129.5, 121.4, 120.1, 6.8, 5.1. MS (ESI): m/z calculated for $C_{12}H_{21}OSi$ ($[M+H]^+$) 209.1356, found 209.1361. Data is consistent with previous report.[2]

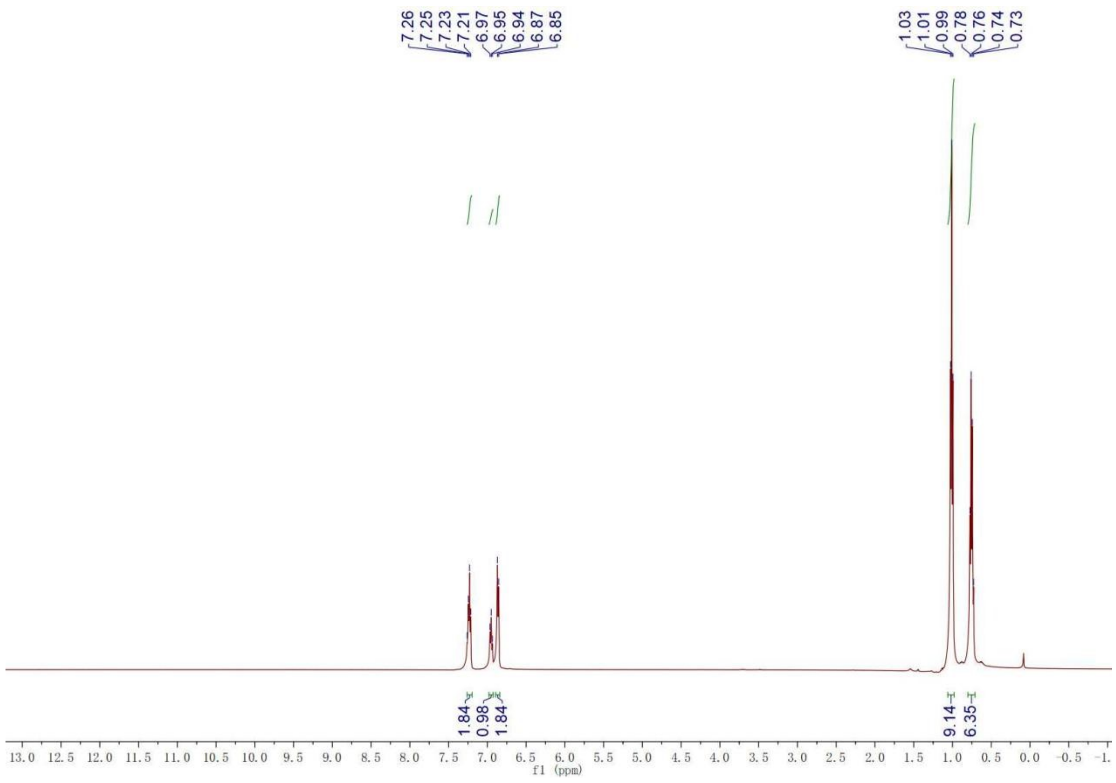

Figure S4. ^{1}H NMR (500 MHz, $CDCl_3$, 298 K) spectrum of triethyl(phenoxy)silane

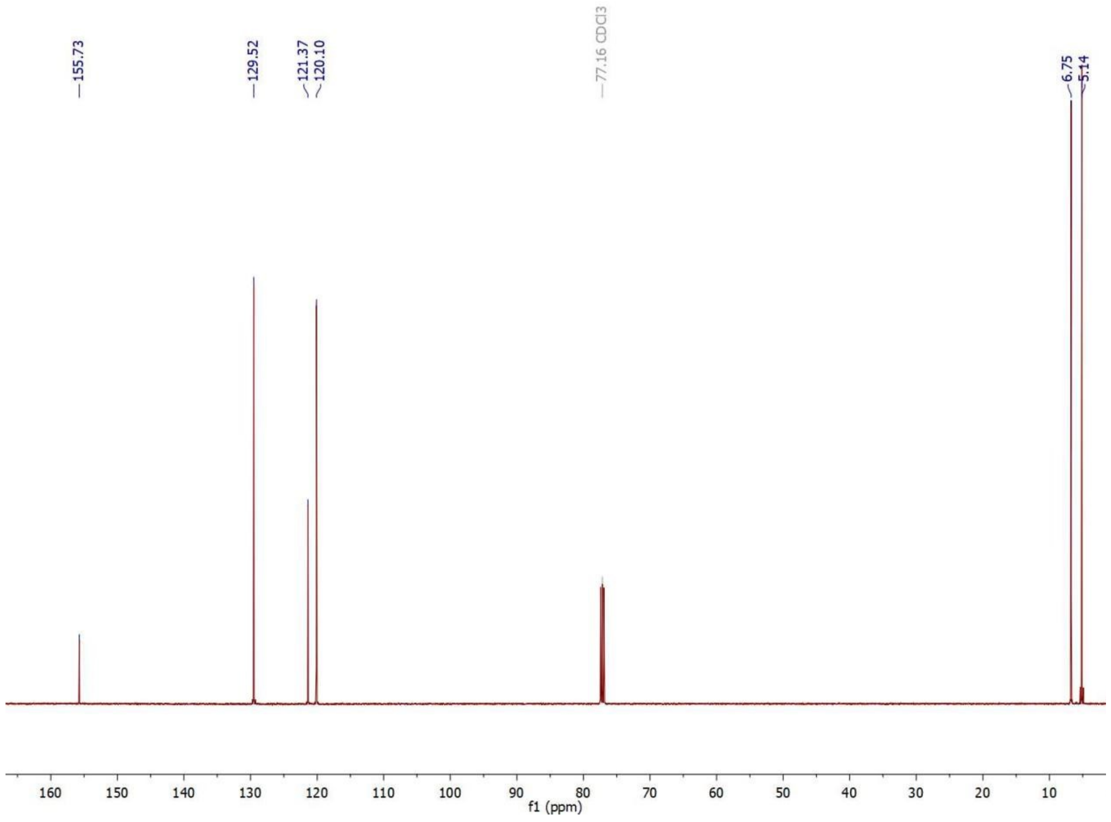

Figure S5. ^{13}C NMR (126 MHz, $CDCl_3$, 298 K) spectrum of triethyl(phenoxy)silane

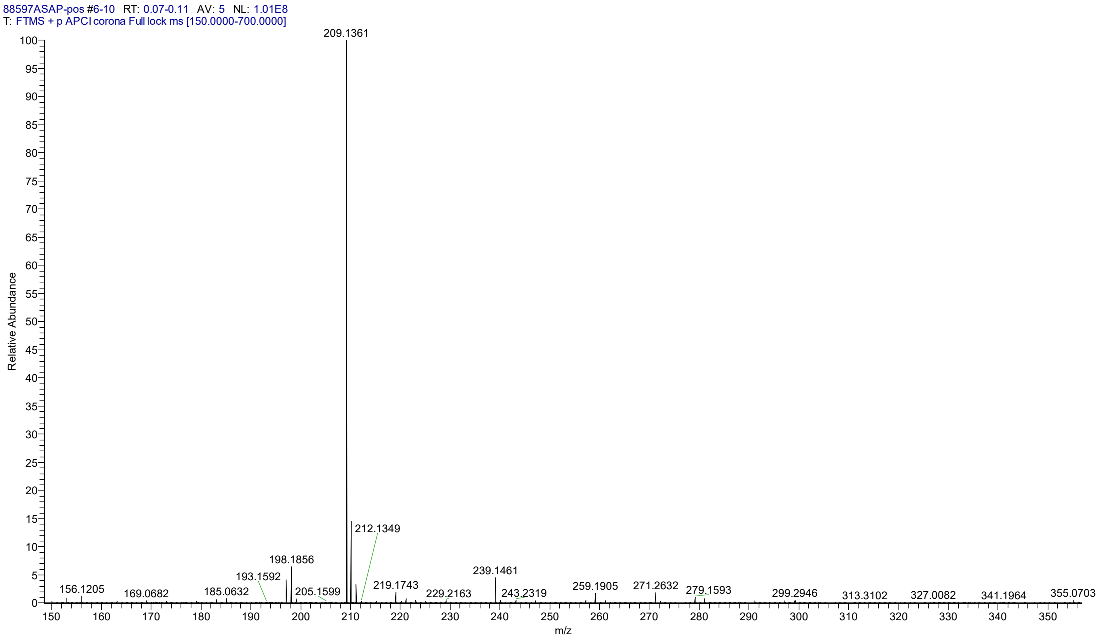

Figure S6. Mass spectrum (ESI+) for triethyl(phenoxy)silane: 209.1361 $[M+H]^+$.

S1.4 Synthesis of triethyl(m-methoxyphenoxy)silane

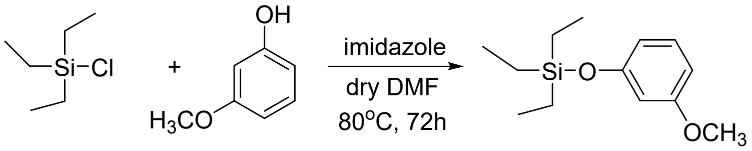

Triethyl(m-methoxyphenoxy)silane was prepared according to previously reported methods.[2] To a solution of imidazole (1200 mg, 17.8 mmol) and *m*-methoxyphenol (740 mg, 654 µL, 5.9 mmol) in dry DMF (9 mL) was added dropwise chlorotriethylsilane (1.5 mL, 8.9 mmol). The reaction was stirred at 80°C for 72 hours. Diethyl ether (150 mL) was then added, and the mixture washed with water (60 mL x 2). The organic phase was dried over anhydrous Na_2SO_4 and concentrated under vacuum. The crude product was purified by column chromatography (hexane:EtOAc, 5:1) to yield the desired compound as pale yellow oil (544 mg, 39%). ^1H NMR (400 MHz, $CDCl_3$, 298 K) δ 7.12 (t, J = 8.0 Hz, 1H), 6.50-6.53 (dd, J = 8.3, 2.3 Hz, 1H, ArH), 6.46 (d, J = 8.0 Hz, 1H, ArH), 6.43 (t, J = 2.2 Hz, 1H, ArH), 3.77 (s, 3H, OCH_3), 1.00 (t, J = 7.9 Hz, 9H, CH_3), 0.72-0.78 (q, J = 8.0 Hz, 6H, CH_2). ^{13}C NMR (126 MHz, $CDCl_3$, 298 K) δ 160.84, 156.92, 129.83, 112.54, 106.93, 106.28, 55.34, 6.77, 5.14. MS (ESI): m/z calculated for $C_{13}H_{23}O_2Si$ ($[M+H]^+$) 239.1462, found 239.1467. Data is consistent with previous report.[2]

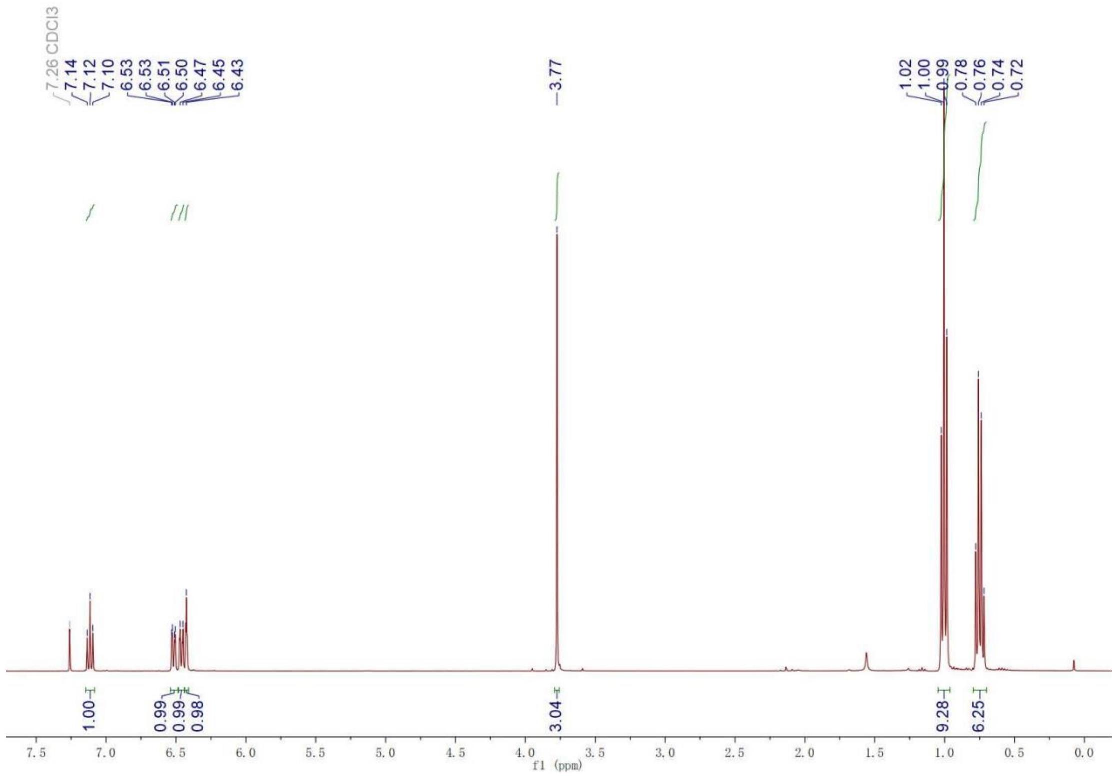

Figure S7. ^{1}H NMR (400 MHz, $CDCl_3$, 298 K) spectrum of triethyl(m-methoxyphenoxy)silane

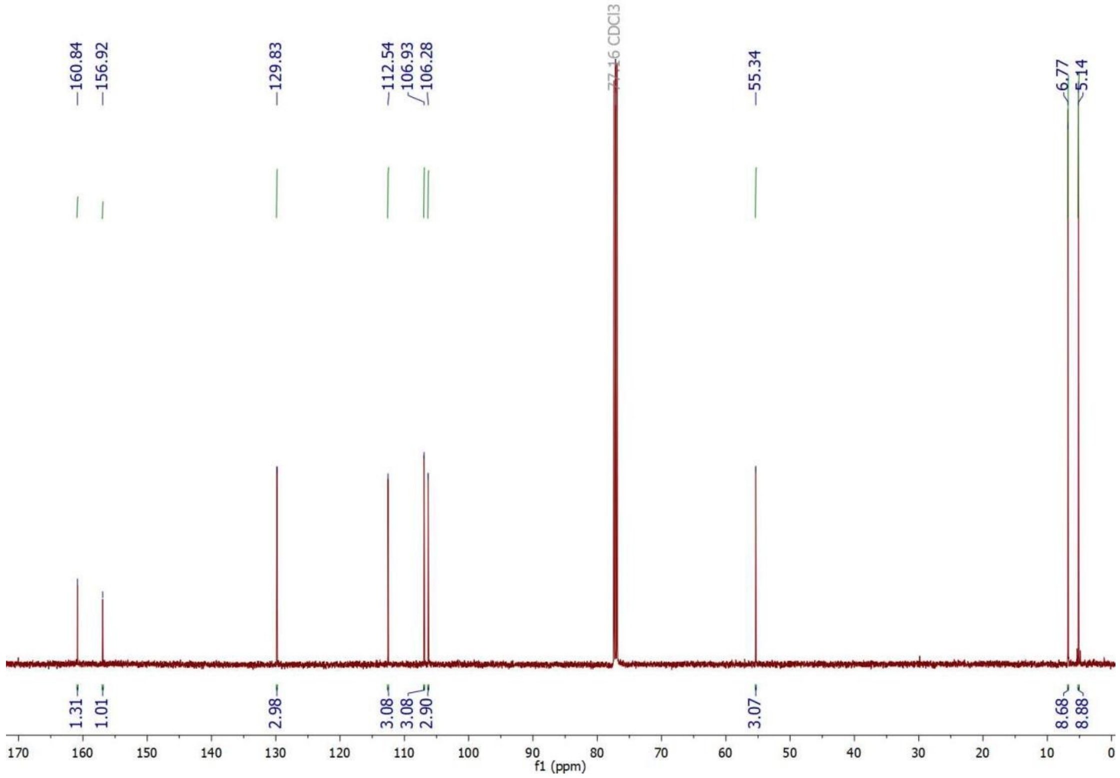

Figure S8. ^{13}C NMR (126 MHz, $CDCl_3$, 298 K) spectrum of triethyl(m-methoxyphenoxy)silane.

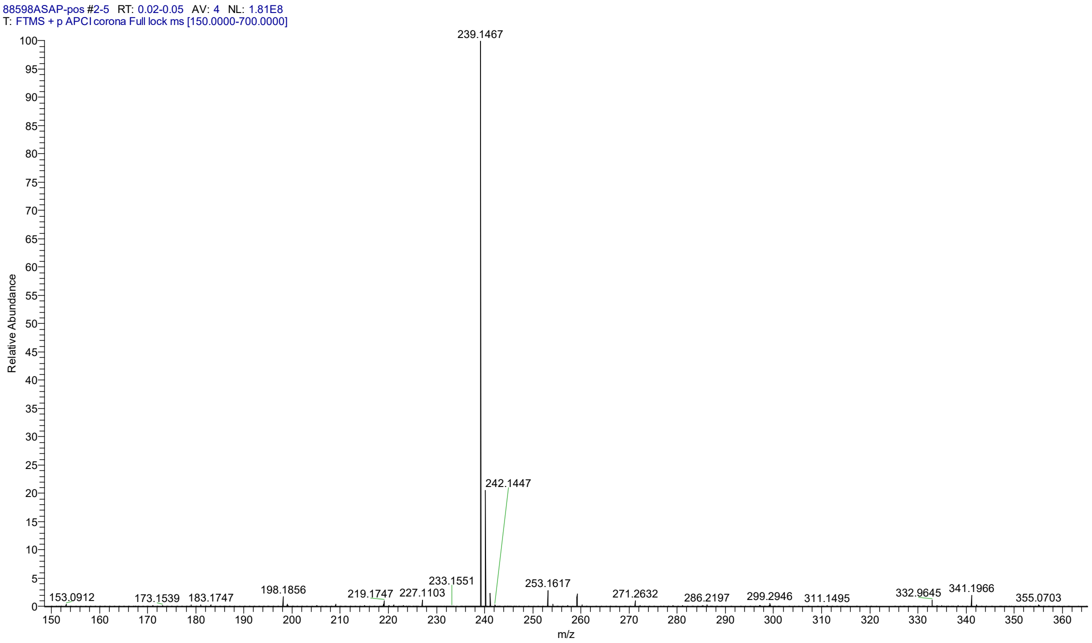

Figure S9. High resolution mass spectrum (ESI+) for triethyl (m-methoxyphenoxy)silane: 239.1467 $[M+H]^+$.

S1.5 Synthesis of triethyl(p-methoxyphenoxy)silane

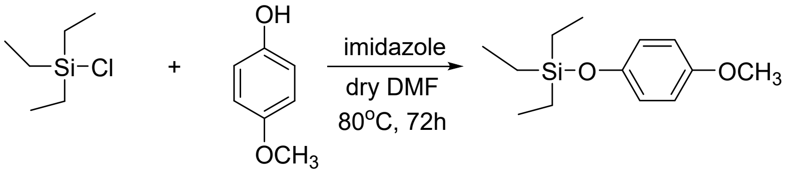

Triethyl(p-methoxyphenoxy)silane was prepared according to previously described methods.[2] To a solution of imidazole (1200 mg, 17.8 mmol) and p-methoxyphenol (740 mg, 5.9 mmol) in dry DMF (9 mL) was injected dropwise chlorotriethylsilane (1.5 mL, 8.9 mmol). The reaction was stirred at 80°C for 72 hours. Diethyl ether (150 mL) was then added to dilute the solution, which was further washed with water (60 mL x 2). The organic phase was dried over anhydrous Na_2SO_4 and concentrated under vacuum. The crude product was purified with column chromatography (hexane:EtOAc = 5:1) to yield the desired compound as pale yellow oil (1035 mg, 74%).^{1}H NMR (400 MHz, chloroform-*d*) δ 6.77 (m, 4H, ArH), 3.76 (s, 3H, OCH_3), 0.99 (t, J = 7.9 Hz, 9H, CH_3), 0.69-0.75 (q, J = 8.4, 7.9 Hz, 6H, CH_2). ^{13}C NMR (101 MHz, chloroform-d) δ 154.19, 149.41, 120.63, 114.60, 55.75, 6.77, 5.04. MS (ESI): m/z calculated for $C_{13}H_{23}O_2Si$ ($[M+H]^+$) 239.1462, found 239.1453. Data is consistent with previous report.[2]

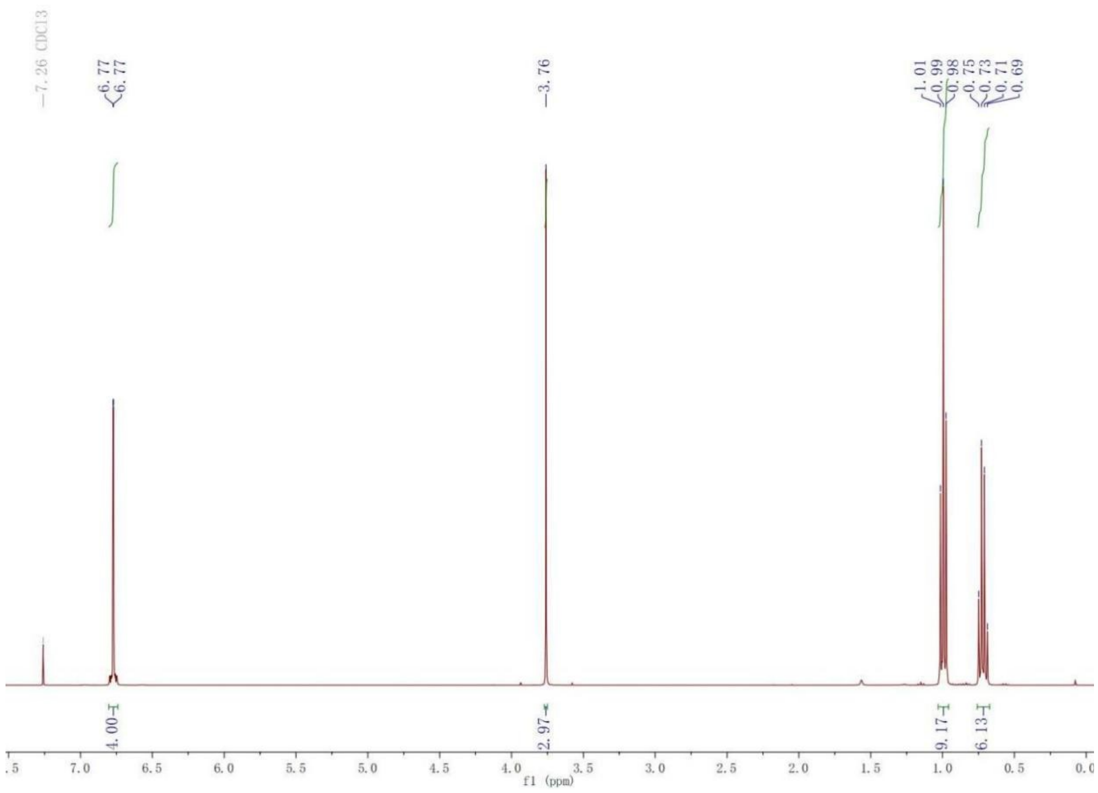

Figure S10. ^{1}H NMR (400 MHz, $CDCl_3$, 298 K) spectrum of triethyl(p-methoxyphenoxy)silane

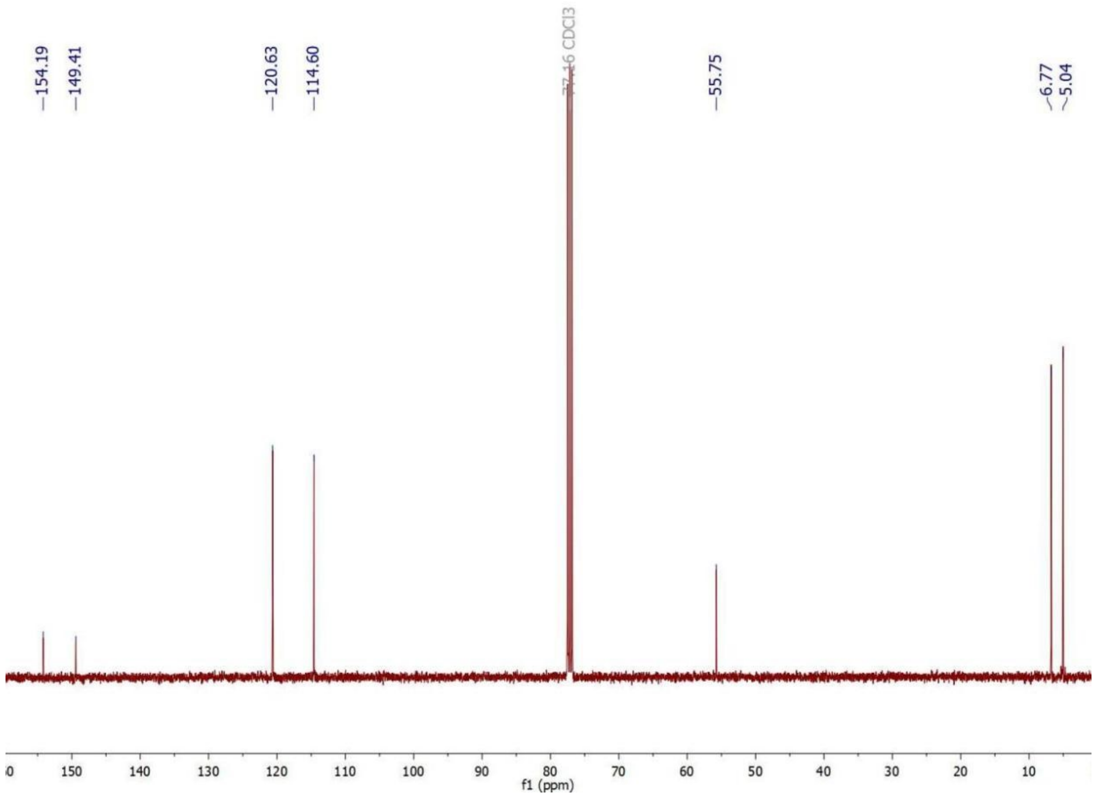

Figure S11. ^{13}C NMR (101 MHz, $CDCl_3$, 298 K) spectrum of triethyl(p-methoxyphenoxy)silane

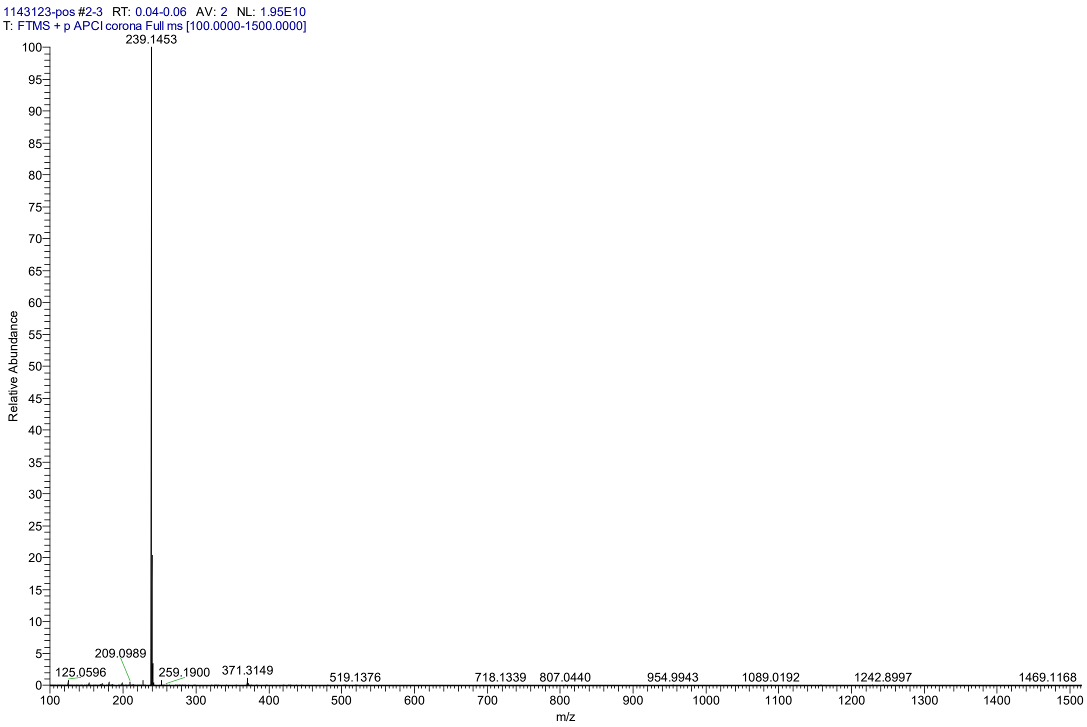

Figure S12. Mass spectrum (ESI+) for triethyl (p-methoxyphenoxy)silane: 239.1453 $[M+H]^+$.

S2. Expression, purification and lyophilization of recombinant silicatein

The expression and purification of TF-Silα and TF-Silα-eGFP were performed according to previously reported procedures.[1] SDS-PAGE was used to ensure the purity of protein eluted from fast protein liquid chromatography (FPLC) (Figure S13). For condensation reaction, TF-Silα solution was exchanged into PBS buffer (Table S1) and lyophilized as reported.[2]

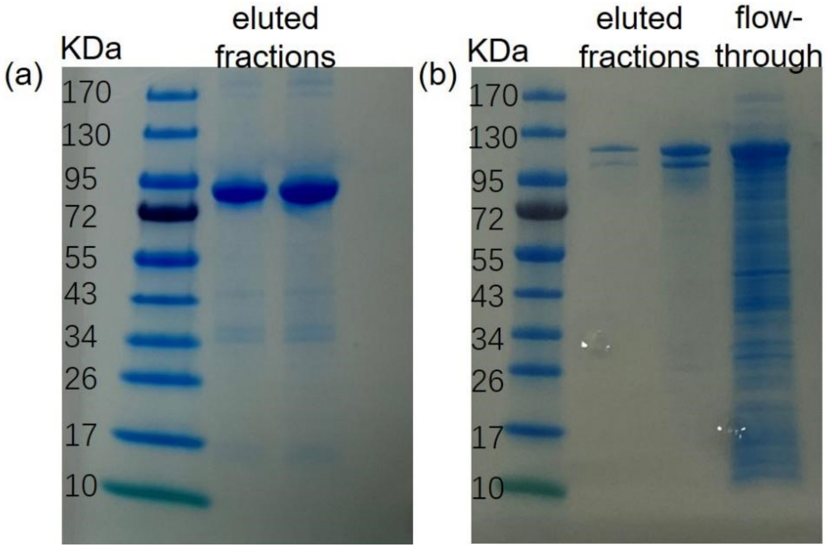

Figure S13. SDS-PAGE of (a)TF-Silα (74.6 KDa) (b)TF-Silα-eGFP (102.8 KDa)

Table S1. Buffers used in this study

Buffer	Contents	pH
Tris buffer	50 mM Tris, 100 mM NaCl	8.5
Strep elution buffer	50 mM Tris, 50 mM biotin, 100 mM NaCl	8.5
PBS buffer	100 mM KH_2PO_4, 100 mM K_2HPO_4, 20 mM KCl	7.0
SDS running buffer	25 mM Tris, 192 mM glycine, 0.1% SDS	8.3

S3. Biomimetic crystallisation of TF-Silα

S3.1 Synthetic procedure for preparation of TF-Silα@Zn-BDC-NH_2

2-Aminoterephthalic acid (0.5 mmol) was added to water and dissolved with 1 M NaOH (1 mL). The pH was adjusted to 8.5 using 1 M HCl before mixed with TF-Silα solution (10 mg in Tris buffer, Table S1) to make a total volume of 20 mL. $Zn(NO_3)_2$ solution (0.5 mmol, 20 mL) was then added and the mixture was stirred at room temperature overnight. The resulting **TF-Silα@Zn-BDC-NH_2** was isolated by centrifugation at 4500 rpm for 10 min, followed by water and methanol washing (25 mL x 2) and then dried under vacuum for 20 min.

S3.2 Synthetic procedure for preparation of Zn-BDC-NH_2

Pure **Zn-BDC-NH_2** was prepared using the same approach as stated in S3.1, except for the absence of enzyme.

S3.3 Synthetic procedure for preparation of TF-Silα-eGFP@Zn-BDC-NH_2

The preparation of **TF-Silα-eGFP@Zn-BDC-NH_2** was same as described in S3.1, except that TF-Silα was replaced by TF-Silα-eGFP (10 mg). The precipitate was recovered by centrifugation (4500 rpm, 10 min) and washed with water (25 mL x 3) before being lyophilized overnight.

S3.4 Synthetic procedure for preparation of TF-Silα-eGFP on MOFs

To prepare enzyme-adsorbed MOFs, pure **ZIF-8** (70 mg) or **Zn-BDC-NH_2** (107 mg) was resuspended in Tris buffer and sonicated for 5 min, before the addition of TF-Silα-eGFP solution (10 mg in Tris buffer) to make a total volume of 40 mL. The mixture was stirred at room temperature overnight and centrifuged at 4500 rpm for 10 min. The solid was washed with water (25 mL x 3) and lyophilized overnight.

S3.5 Synthetic procedure for preparation of TF-Silα@ZIF-8

TF-Silα solution (10 mg) was mixed with 2-methyl-imidazole solution (3.2 mmol) using Tris buffer (pH 8.5, Table S1) as solvent, and the total volume was adjusted to 20 mL before the addition of $Zn(NO_3)_2$ aqueous solution (0.8 mmol, 20 mL). The mixture was stirred at room temperature for 0.5 hour, and the precipitate was recovered by centrifugation at 4500 rpm for 10 min. The solid was washed twice with water (25 mL) and methanol (25 mL) and dried under vacuum.

S3.6 Synthetic procedure for preparation of ZIF-8

ZIF-8 was synthesized in the same method as stated in S3.5, except that no enzyme was introduced in the system and the reaction time was elongated to 2 days.

S3.7 Synthetic procedure for preparation of TF-Silα-eGFP@ZIF-8

The synthesis of **TF-Silα-eGFP@ZIF-8** was the same as described above in S3.5, except for TF-Silα was replaced by TF-Silα-eGFP (10 mg). After 0.5 h reaction, the mixture was centrifuged (4500 rpm, 10 min) and the precipitates was washed 3 times with water (25 mL) before being lyophilized overnight.

S3.8 Protein quantification

The protein solutions listed in Table S2 were prepared from 2 $mg.mL^{-1}$ BSA standard, using H_2O as diluent. To each well of a 96-well microtitre plate, 5 µL of the solutions containing each protein concentration was added before being mixed with 250 µL Coomassie dye solution (warmed up to room temperature before use). Absorbance at 595 nm (Y) was measured and plotted against protein concentrations (X). The resulting calibration curve was used to determine unknown protein concentration, with the linear range for BSA from 1 to 100 $\mu g.mL^{-1}$.

For enzyme@MOF composites, the reaction mixture was centrifuged (4500 rpm, 15 min) and 5 µL of the supernatant was added to the plate and mixed with 250 µL Coomassie dye. Encapsulation efficiency (EE) and loading content (LC) are defined as below[4]:

$$\text{Encapsulation efficiency (EE\%)} = \frac{m_{(enzyme)} - cV}{m_{(enzyme)}} \times 100\%$$

Equation 1: Calculation of encapsulation efficiency; where $m_{(enzyyme)}$(mg) refers to total enzyme amount added at the start of the experiment, c(mg/mL) refers to enzyme concentration in the supernatant, and V(mL) indicates supernatant volume.

$$\text{Loading content (LC\%)} = \frac{m_{(enzyme\ in\ enzyme@MOFs)}}{m_{(enzyme@MOFs)}} \times 100\%$$

Equation 2**:** Calculation of loading content; where $m_{(enzyme\ in\ enzyme@MOFs)}$ (mg) refers to the mass of enzyme in the MOF, $m_{(enzyme@MOFs)}$ (mg) indicates the weight of enzyme@MOFs

Table S2. BSA concentrations used in Bradford assay

Tube	Standard volume (μL)	Source of standard	Diluent volume (μL)	Final protein concentration (μg/mL)
1	20	2 mg/mL stock	0	2000
2	2	2 mg/mL stock	38	100
3	20	Tube 2	20	50
4	20	Tube 3	20	25
5	20	Tube 4	20	12.5
6	4	Tube 2	36	10
7	20	Tube 6	20	5
8	4	Tube 6	36	1

The encapsulation efficiency and loading content of different **enzyme@MOF** composites were calculated and are listed in Table S3. Results are averaged from at least 3 measurements.

Table S3. Encapsulation performance of enzyme@MOF composites. The variance indicates standard deviation from different batches.

Enzyme@MOF	EE(%)	LC(%)
TF-Silα@ZIF-8	(86.5±3.6)%	(14.4±0.6)%
TF-Silα@Zn-BDC-NH_2	(95.8±3.0)%	(9.6±0.8)%
TF-Silα-eGFP@Zn-BDC-NH_2	(97.9±1.2)%	(9.6±0.4)%

S4. Characterization of TF-Silα@MOF composites

S4.1 Fourier transform infrared spectroscopy (FTIR) analysis

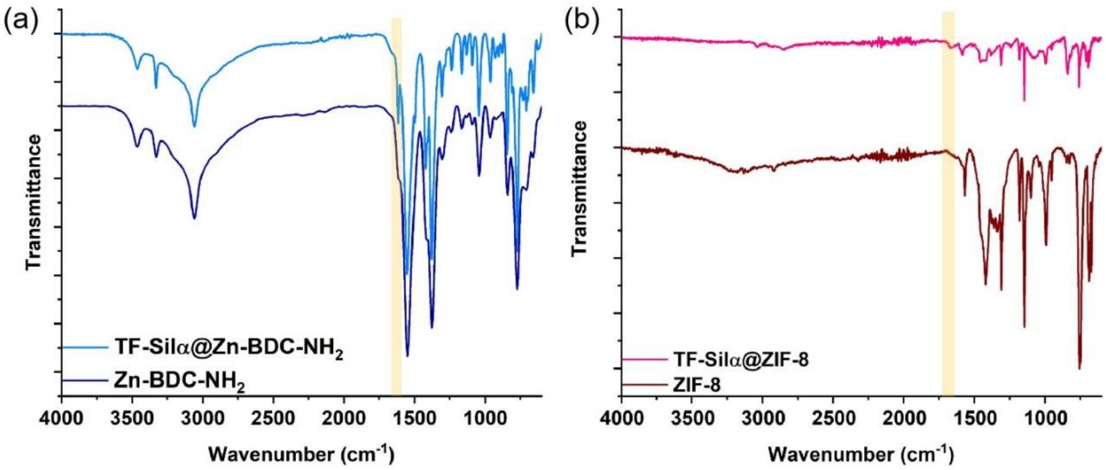

Figure S14. FTIR spectra of (a) **TF-Silα@Zn-BDC-NH_2**(light blue), **Zn-BDC-NH_2**(mid blue) and (b) **TF-**

Silα@ZIF-8(pink), **ZIF-8**(red). The amide I band is highlighted with a yellow box.

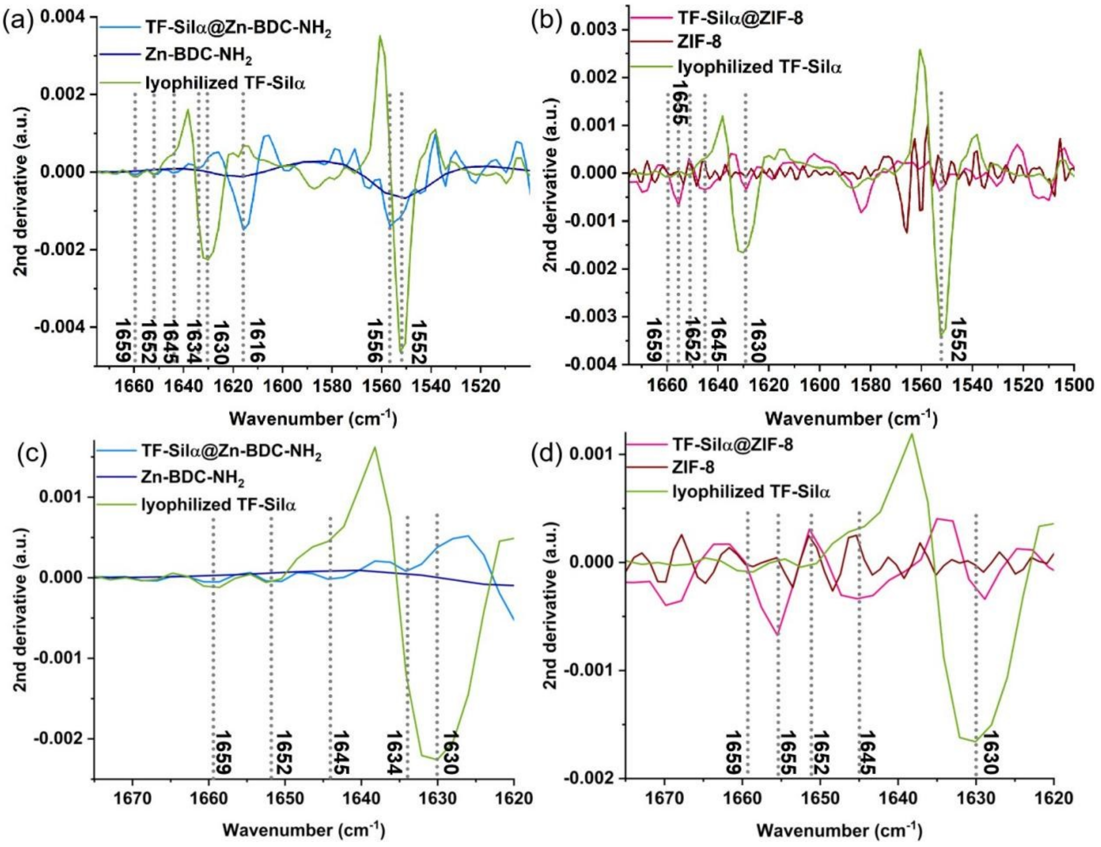

Figure S15. Second derivative FTIR spectra of (a) **TF-Silα@Zn-BDC-NH$_2$**(light blue), **Zn-BDC-NH$_2$**(blue) and lyophilized TF-Silα(green); (b) **TF-Silα@ZIF-8**(red)**, ZIF-8** (brown) and lyophilized TF-Silα(green). Magnified spectra of amide I region for (c) **TF-Silα@Zn-BDC-NH$_2$**(light blue), **Zn-BDC-NH$_2$**(blue) and lyophilized TF-Silα(green); (d) **TF-Silα@ZIF-8**(red)**, ZIF-8** (brown) and lyophilized TF-Silα(green).

Second derivative analysis of the FTIR spectra was conducted to deconvolute the amide I region and assess the structural integrity of TF-Silα after MOF encapsulation. As TF-Silα is a recombinant protein that is difficult to crystallize, lyophilized TF-Silα was used as a reference for structural comparison. As shown in Figure S15, the amide I band (1600-1700 cm^{-1}) of TF-Silα comprises contributions from α-helices (1659, 1652 and 1645 cm^{-1}) and β-sheets (1630 cm^{-1}).[5] The protein also exhibits a sharp amide II band at 1552 cm^{-1}, which is characteristic of α-helical structures. For **TF-Silα@Zn-BDC-NH$_2$** (Figure S15a,c), the α-helical features remained intact, but the amide II band was blueshifted by ~4 cm^{-1}. This shift is tentatively attributed to the confinement of TF-Silα within the framework, which reduces hydrogen bonding between protein N-H groups and surrounding water molecules thereby resulting in a tighter N-H bond and an increased vibrational frequency.[6] For **TF-Silα@ZIF-8** (Figure S15b,d), the β-sheet peak at 1630 cm^{-1} was retained, while the α-helical peaks were redshifted by 4 - 7 cm^{-1}. This shift likely reflects a modest weakening of C=O bonds due to interaction with the framework.[6] In general, the observed spectral shifts are relatively minor, and both composites retain the characteristic secondary structures of TF-Silα, indicating the enzyme structural integrity is largely maintained upon MOF encapsulation.

S4.2 Circular Dichroism (CD)

CD spectroscopy was performed in Tris Buffer (pH 8.5) on an Applied-Photophysics Chirascan qCD spectrometer using a 0.1 mm path-length cuvette. Experiments were performed at 25 °C, maintained using a Quantum Northwest TC125 temperature controller. Measurements were background subtracted (blank buffer) in an identical cuvette, and concentrations were selected to maintain an average absorbance below 1.2 a.u.. The experimental concentrations for BDC-NH_2 and 2-mIm when preparing enzyme@MOFs are 25 mM and 160 mM, respectively. These concentrations were further diluted to 6.25 mM and 8 mM to acquire CD spectra with less interference and obtain smoother absorbance data.

Data was collected in triplicate and averaged between 280 - 190 nm with a scan width of 1 nm at 0.5 seconds per data point.

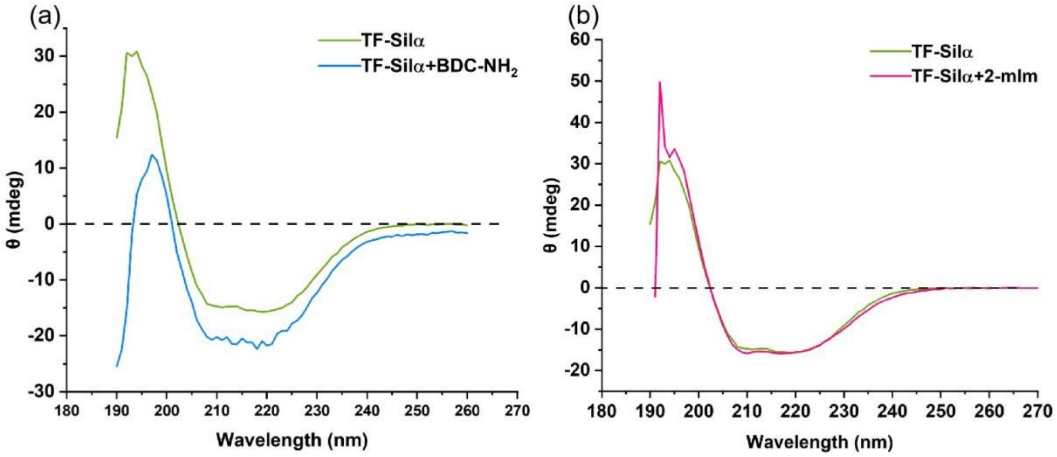

Figure S16. Circular dichroism spectra of TF-Silα (green) in Tris buffer (Table S1) and a mixture of (a) TF-Silα with 2-aminoterephthalic acid (BDC-NH_2, 6.25 mM) at pH 8.5; (b) TF-Silα with 2-methyl imidazole (2-mIm, 8mM) at pH 8.5. The protein concentration was set at 1.3 $mg.mL^{-1}$ for all the measurements. Background absorbance from Tris buffer was subtracted from all samples. The curves were averaged from three measurements.

To further assess the structural integrity of encapsulated TF-Silα, circular dichroism (CD) spectroscopy was performed on both the free enzyme and the enzyme mixed with each of the ligands employed in biomimetic crystallization. As shown in Figure S16, free TF-Silα exhibited the characteristic signatures of an α-helical structure, with a positive band at 193 nm and negative bands at 208 and 222 nm, consistent with previous reports.[1, 7] Upon mixing with either ligand, the α-helical features were preserved, indicating that TF-Silα maintained its folded conformation under the tested conditions(Figure S16a,b). The reduction in ellipticity intensity in the presence of BDC-NH_2 (Figure S16a) is attributed to the strong absorbance of this aromatic ligand in the far-UV region, which decreases transmitted light and consequently reduces the observed signal. CD measurements were also attempted with higher, prenucleation concentration of ligands, but the excessive UV absorbance led to detector saturation and prevented reliable data acquisition. Attempts to record CD spectra of TF-Silα in the presence of zinc ions were unsuccessful, due to the formation of precipitate. Collectively, these findings support that TF-Silα remains structurally

intact during Zn-BDC-NH_2 and ZIF-8 nucleation.

S4.3 Thermogravimetric analysis (TGA)

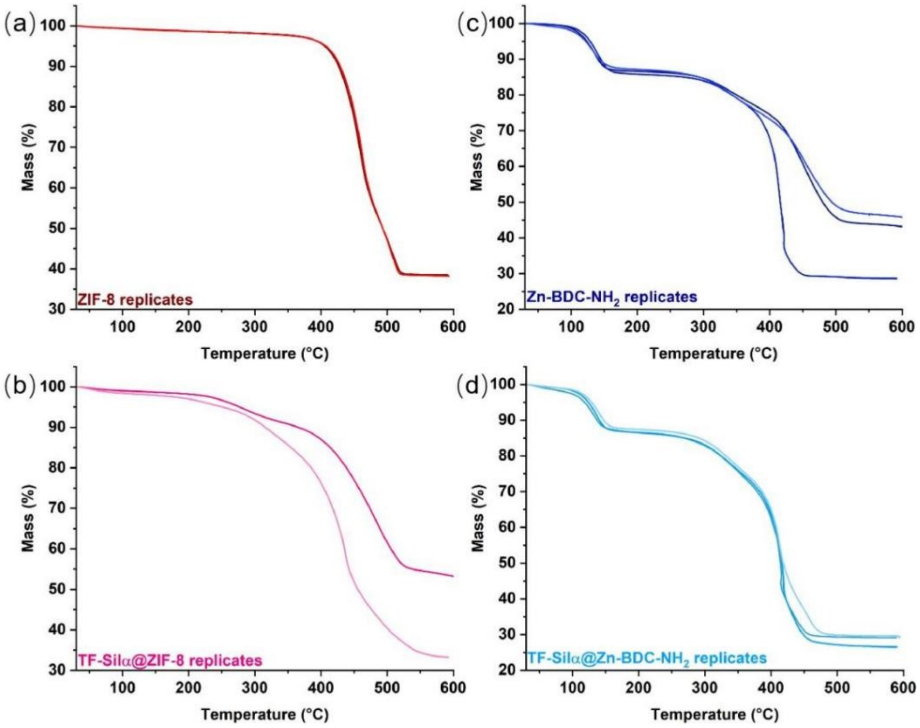

Figure S17. TGA replicates of (a) **ZIF-8** (b) **TF-Silα@ZIF-8** (c) **Zn-BDC-NH_2** and (d) TF-Silα@Zn-BDC-NH_2.

TGA was performed for each of the pure **MOF** and **enzyme@MOF** composites in order to study their thermal degradation patterns. As shown in Figure S15 a,b, **ZIF-8** went through a 2-step degradation whereas the 3 steps were observed for **TF-Silα@ZIF-8**. In the first stage (30 - 200 °C), both pure and enzyme-incorporated MOF lost around 2% mass due to solvent evaporation. Between 200 and 320 °C, however, no mass loss was observed for **ZIF-8**, whereas **TF-Silα@ZIF-8** lost 6.6% weight on average. Since this temperature range is lower than the **ZIF-8** decomposition point (450-500 °C)[8], this mass loss is attributed to protein degradation. At temperatures higher than 400 °C, a large mass loss was observed for both materials due to the MOF decomposition.

Similarly, both **Zn-BDC-NH_2** and **TF-Silα@Zn-BDC-NH_2** went through a 3-step weight reduction. During the first step between 40 and 200 °C, both materials lost 12%-14% of their weight, involving loss of water. In the second stage (300 - 400 °C), the pure MOF lost 12.2% weight on average, this we attribute to linker degradation (the decomposition point of 2-aminoterephthalic acid is 342 °C[9]). Meanwhile, the **enzyme@MOF** lost an average mass of 20.5%, this 8.3% greater mass loss was assigned to protein degradation. At temperatures greater than 400 °C significant mass loss was observed for both materials as the MOF decomposed. Deviations in the TGA curves for replicate samples are observed in the high temperature region (400 - 600 °C). This is attributed to variation in protein and solvent loading between batches. In particular, variation in protein loading introduces different levels of defects in the frameworks, consequently affecting the thermal stability of the MOF scaffold. Notably, previous research has demonstrated that increased protein

loading can lead to a reduction in MOF decomposition temperature.[10] Therefore, TGA data provides an estimation of the enzyme@MOF composition and is primarily used here to support evidence of successful protein incorporation.

S4.4 Protein Localisation via Energy Dispersive Spectroscopy (EDX) and Confocal Laser Scanning Microscopy (CLSM)

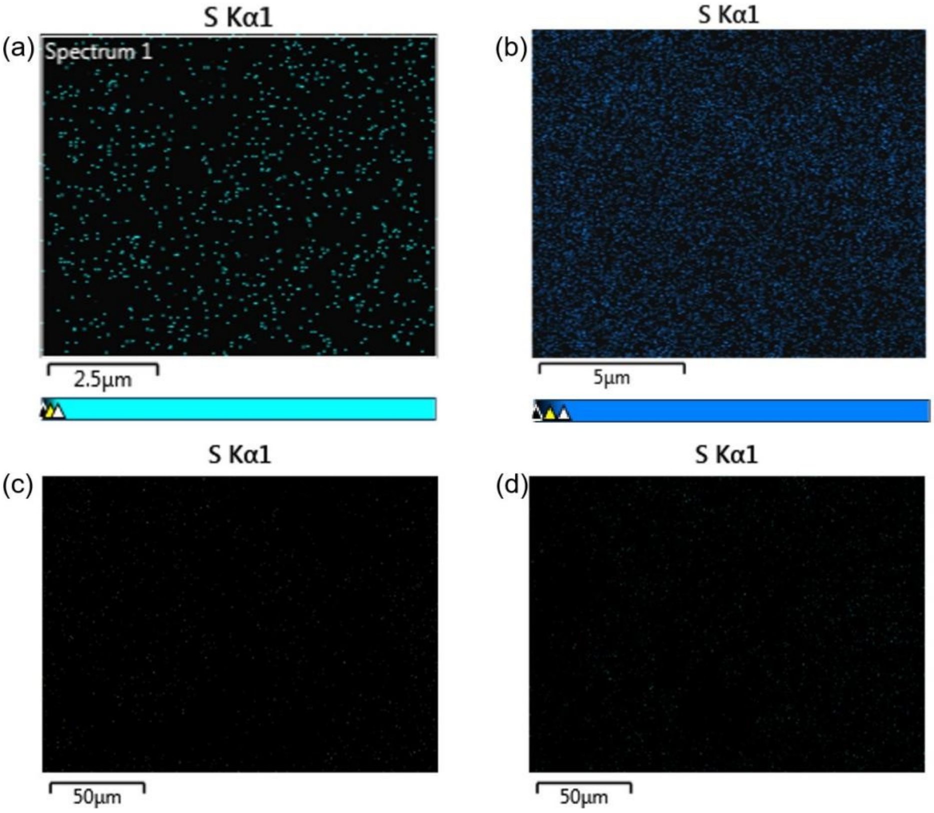

Figure S18. EDX images presenting X ray fluorescence of sulfur observed in (a) **TF-Silα@ZIF-8** (b) **TF-Silα@Zn-BDC-NH$_2$** (c) **ZIF-8** and (d) **Zn-BDC-NH$_2$**

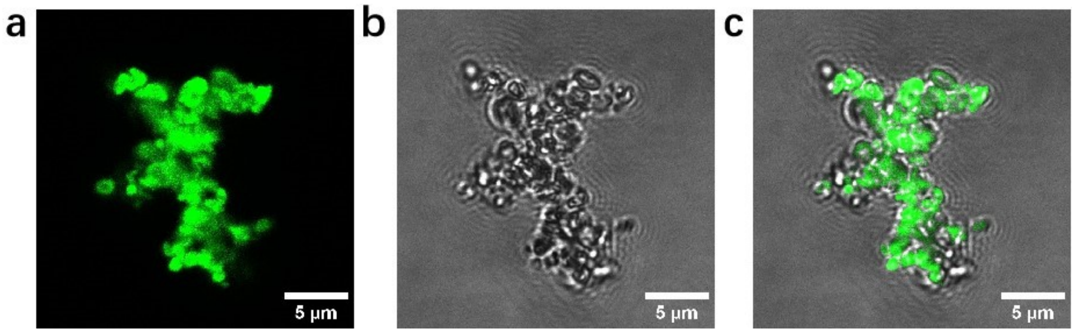

Figure S19. Confocal laser scanning micrograph of **TF-Silα-eGFP@ZIF-8** (a) dark field excited at 488 nm (b) bright field (c) merged.

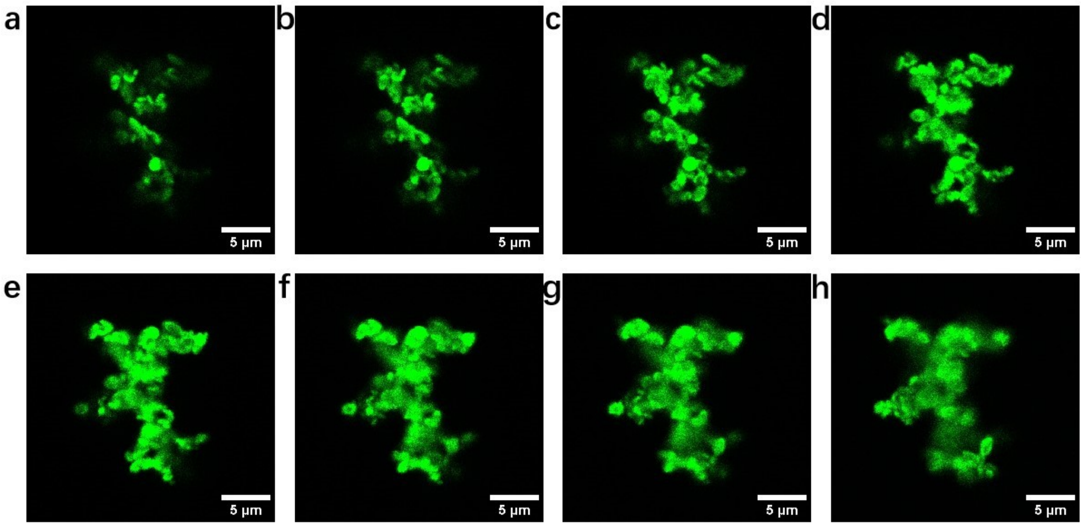

Figure S20. Confocal Z-stack images of **TF-Silα-eGFP@ZIF-8** particles. Figure (a) to (h) represent 8 of the 16 slices of the sample from top to bottom (a to h correspond to z = 2,4,6,8,10,12,14,16 respectively), and the distance between adjacent slices is 0.3 µm.

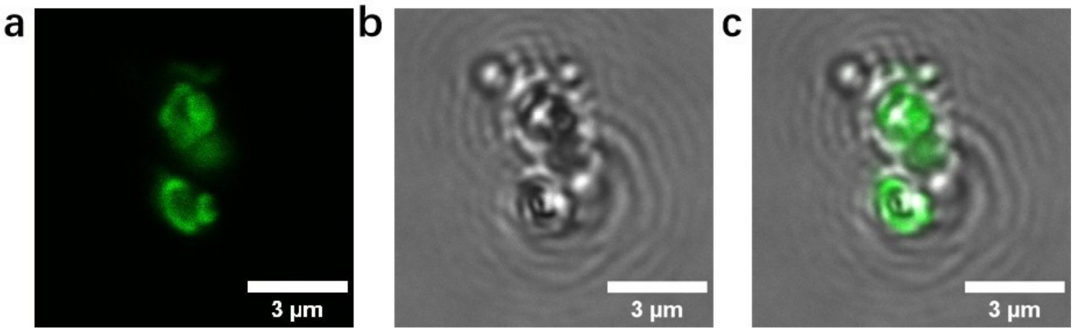

Figure S21. Confocal laser scanning micrograph of **TF-Silα-eGFP** on **ZIF-8** (a) dark field excited at 488 nm (b) bright field (c) merged.

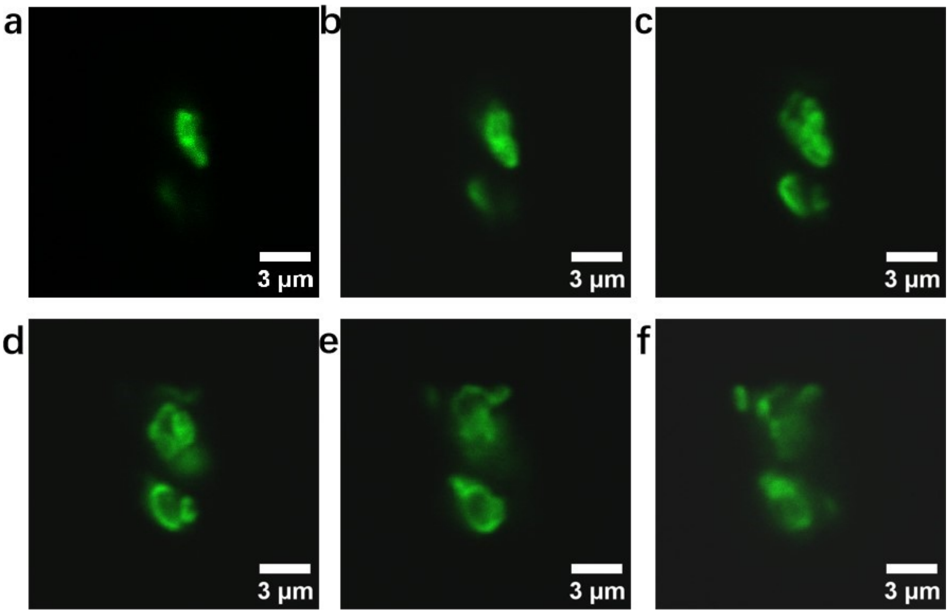

Figure S22. Confocal Z-stack images of **TF-Silα-eGFP** on **ZIF-8** particles. Figure (a) to (h) represent 6 of the 18 slices of the sample from top to bottom (a to h correspond to z = 3,6,9,12,15,18 respectively), and the distance between adjacent slices is 0.3 μm. In comparison to images of **TF-Silα-eGFP@ZIF-8** where fluorescence is more uniformly distributed the **TF-Silα-eGFP** on **ZIF-8** images show void areas indicative of protein aggregation on the surface of particles / aggregates.

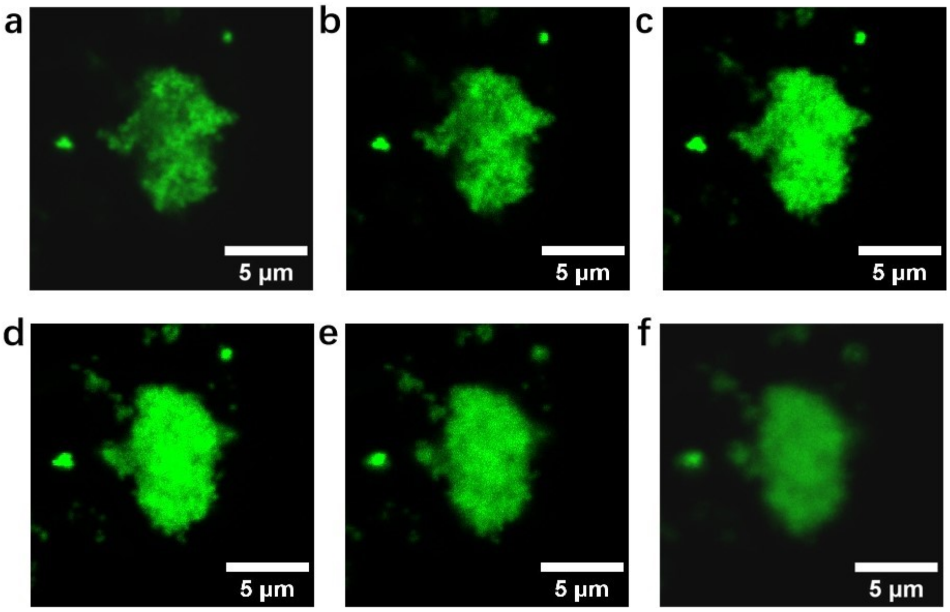

Figure S23. Confocal Z-stack images of **TF-Silα-eGFP@Zn-BDC-NH$_2$** particles. Figure (a) to (h) represent 6 of the 14 slices of the sample from top to bottom (a to h correspond to z = 2,4,6,8,10,12,14, respectively), and the distance between adjacent slices is 0.3 μm

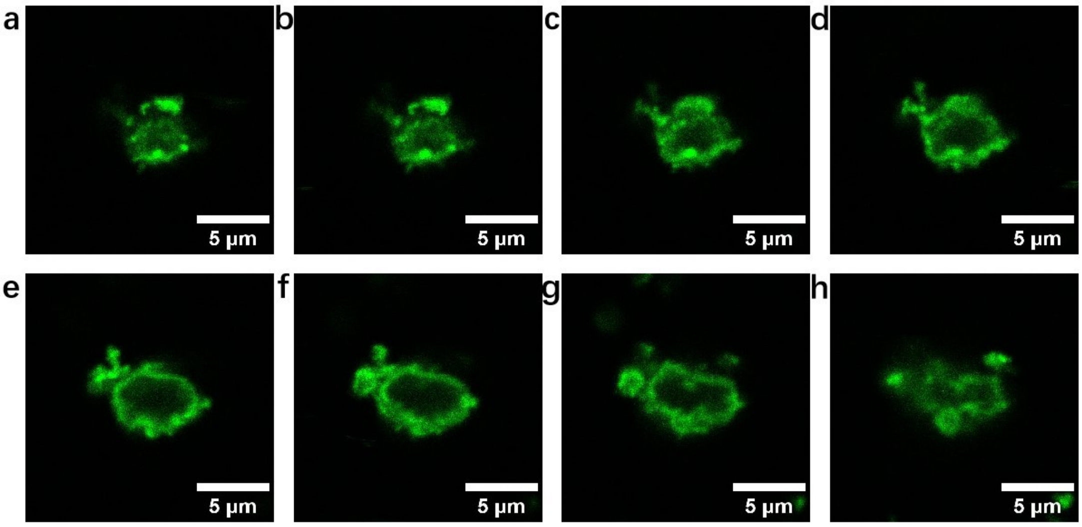

Figure S24. Confocal Z-stack images of **TF-Silα-eGFP** on **Zn-BDC-NH$_2$** particles. Figure (a) to (h) represent 8 of the 21 slices of the sample from top to bottom (a to h correspond to z = 1,3,6,9,12,15,18,21, respectively), and the distance between adjacent slices is 0.3 µm. In comparison to images of **TF-Siα-eGFP@Zn-BDC-NH$_2$** where fluorescence is uniformly distributed the **TF-Silα-eGFP** on **Zn-BDC-NH$_2$** images show void areas indicative of protein aggregation on the surface of particles / aggregates.

S4.5 Electron diffraction (ED)

For the three dimensional ED experiment, the sample MOF was dispersed by sonication in diethyl ether and dropped onto a copper-supported holey carbon TEM grid which was allowed to evaporate dry. This was flash-frozen in liquid nitrogen and loaded at ~115 K via a Gatan Elsa cryo holder into a Rigaku XtaLAB Synergy-ED electron diffractometer, operated at 200 kV and equipped with a Rigaku HyPix-ED hybrid pixel array area detector. Data for three crystallites (see Table S4) appearing as slabs were collected at 100(5) K using selected area continuous rotation electron diffraction with a selected area aperture of 2 µm apparent diameter. Data were collected using CrysAlisPRO (version 171.44.78a 64-bit, release 25-10-2024)[11], then individually indexed and integrated, then merged to a limit of d = 0.8 Å and scaled (see Table S5) into a single dataset using CrysAlisPRO (version 1.171.44.88a)[11] The structure was solved using ShelXT[12] and refined in the kinematic approximation using Olex2.refine as implemented in Olex2 (version 1.5-ac7-013, compiled 2025.01.02 svn.rf662f148 for Rigaku Oxford Diffraction, GUI svn.r7109)[13, 14], using published scattering factors[15]. An extinction correction was applied to broadly account for the impact of multiple diffraction with further omission of particularly outlying reflections in the final stages of the refinement. All protons were refined in the presence of a distance similarity restraint for chemically equivalent groups and C-H hydrogen atoms were further geometrically restrained, all with riding isotropic displacement parameters. A global rigid bond restraint was employed to improve the physical sense of the anisotropic displacement parameters for all non-hydrogen atoms with the additional of an isotropicity restraint on O16. Experimental and refinement information are contained within the deposited CIF along with structure factors and an embedded .RES file, while the structure is deposited in the CSD with deposition code CCDC 2418698.

Table S4. Experimental crystal details

	Crystal 1	Crystal 2	Crystal 3
a (Å)	9.516(2)	9.515(2)	9.502(5)
b (Å)	6.4479(7)	6.4481(10)	6.4295(12)
c (Å)	14.8831(16)	14.906(2)	14.985(3)
β (°)	95.055(17)	95.233(13)	95.21(3)
V ($Å^3$)	909.6(2)	910.7(3)	911.8(6)
~Crystal size (µm)	2×1×0.3	1.5×1×0.5	2×2×1
h_{min}, h_{max}	-14, 14	-13, 13	-13, 13
k_{min}, k_{max}	-9, 9	-8, 8	-8, 8
l_{min}, l_{max}	-23, 23	-23, 23	-23, 23
Measured reflections	7491	8476	5984
Completeness (0.80 Å)	0.7617	0.763	0.6041
R_{int} (0.80 Å, max)	0.1581, 0.1894	0.1450, 0.1782	0.1801, 0.2207

Table S5. Experimental data details

Crystal data	
Chemical formula	$ZnC_8H_9NO_6$
M_r	280.55
Crystal system, space group	Monoclinic, $P2_1/c$
Temperature (K)	100(5)
a, *b*, *c* (Å)	9.5783(15), 6.4427(6), 14.9087(12)
β (°)	95.23(18)
V (Å^3)	909.53(18)
Z	4
Radiation type	Electron, λ = 0.02510 Å
Data collection	
h_{min}, h_{max}	−11, 11
k_{min}, k_{max}	−8, 8
l_{min}, l_{max}	−18, 18
Diffractometer	XtaLAB Synergy-ED, HyPix-ED, electron source at 200keV
Merging	*CrysAlis PRO* 1.171.44.78a (Rigaku Oxford Diffraction, 2024) SCALE3 ABSPACK scaling and empirical correction using spherical harmonics.
measured, independent, observed [$I \geq 2u(I)$] reflections, completeness (0.80 Å)	12093, 1670, 1490, 0.8965
R_{int}	0.202
$(\sin \theta/\lambda)_{max}$ (Å^{-1})	0.626
Refinement	
R_1, wR_2 ([$F^2 > 2\sigma(F^2)$])	0.1437, 0.2873
R_1, wR_2 (all)	0.1598, 0.2966
GoF(*S*) (inc., excl. restraints)	0.9889, 1.0358
data, parameters, restraints	1670, 167, 146
$\Delta\phi_{max}$, $\Delta\phi_{min}$ (as reported by Olex2.refine, AC07-013)	0.814, −0.775

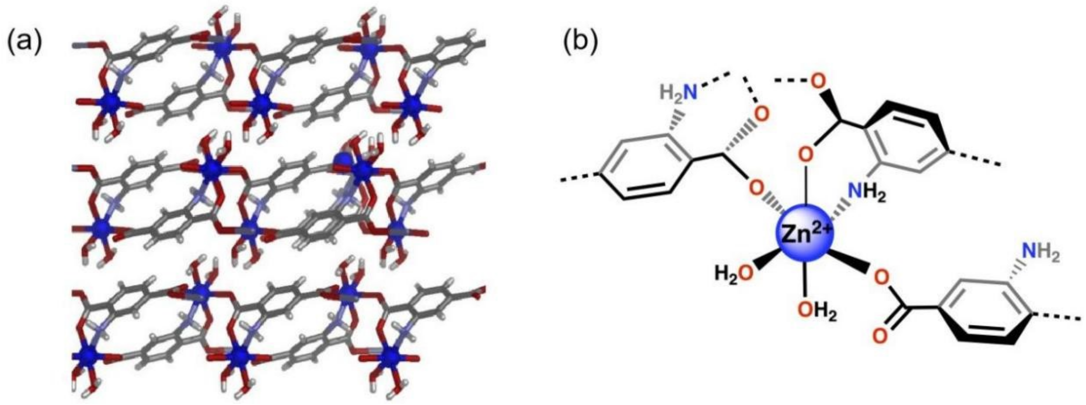

Figure S25. (a) Schematic presentation of crystal structure of **TF-Silα@Zn-BDC-NH$_2$** when synthesized at pH 8.5, derived from ED and PXRD analysis[16]. (b) Schematic representation of zinc(II) ion coordination sphere.

S4.6 Powder X-ray diffraction (PXRD)

Data were collected using a Phillips X'pert in Bragg-Brentano geometry, with fixed divergence slits (0.125° + 0.25°), antiscatter slits and an X'celerator 128 channel strip detector. Data were collected using X'pert data collector v2.0e. Samples were prepared manually by depositing powder onto zero background silicon plates.

Peak hunting and unit cell indexing was performed using TOPAS software.[17]Le Bail profile analysis was performed using JANA2020 software.[18] The lack of a beam knife with this diffractometer setup results in large peak asymmetry at low angle, which is apparent in the data

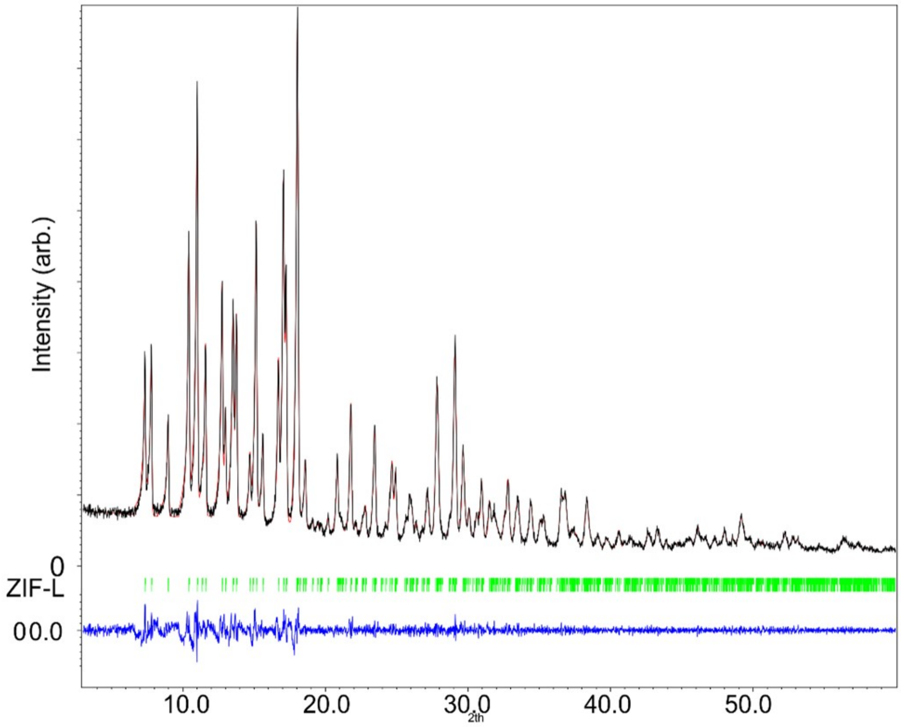

Figure S26. Le bail profile fitting for **ZIF-8** biomimetic preparation in the absence of protein. Black = observed data, red = calculated profile, green = allowed Miller indices, blue = Obs-Calc difference. Unit cell matches that of published **ZIF-L** CCDC 1509273

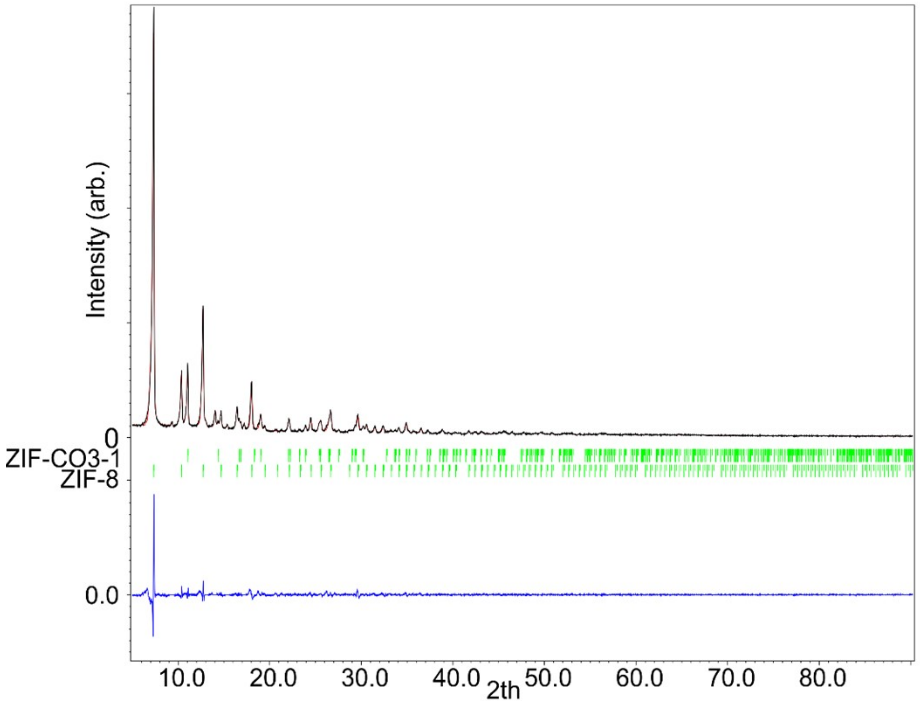

Figure S27. Le bail profile fitting for **TF-Silα@ZIF-8**. Black = observed data, red = calculated profile, green = allowed Miller indices, blue = Obs-Calc difference. Sample is a mixture of **ZIF-CO_3-1** and **ZIF-8**. Unit cell matches that of published **ZIF-CO_3-1** and **ZIF-8** CCDC 1032088 and 602542, respectively

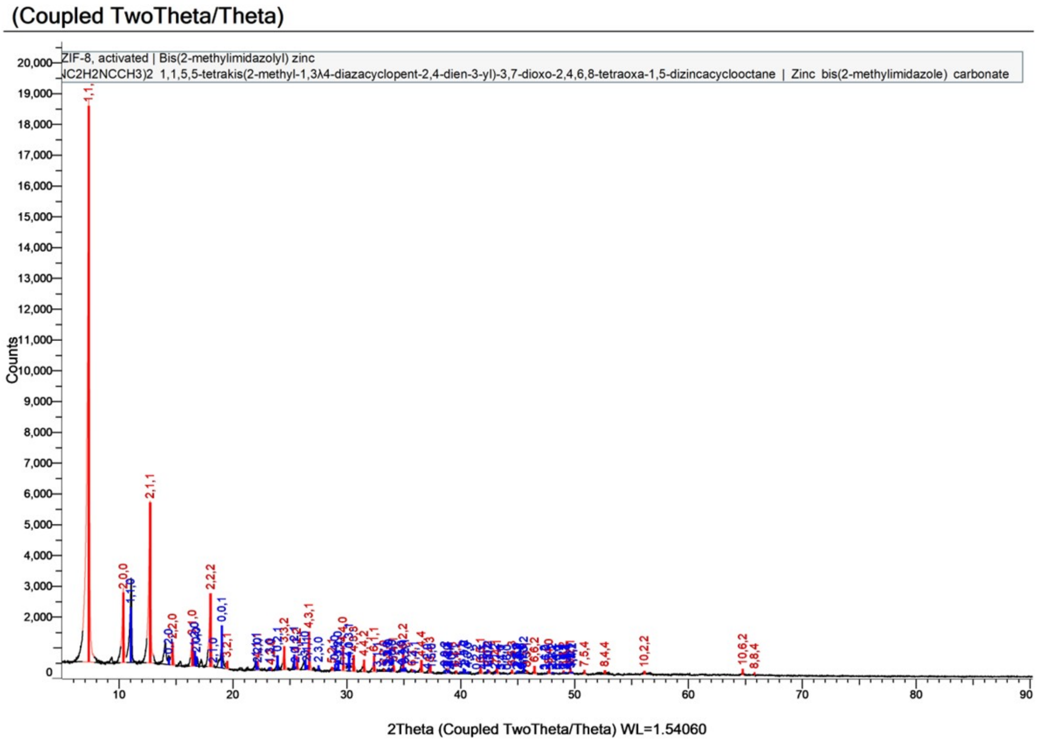

Figure S28. Semi-quantitative analysis of **TF-Silα@ZIF-8** fitting. **TF-Silα@ZIF-8** consists of 78.5% **ZIF-8** and 21.5% **ZIF-CO_3-1**, based on relative intensity ratios calculation. The analysis was performed withBruker EVA, Bruker AXS 2025software.

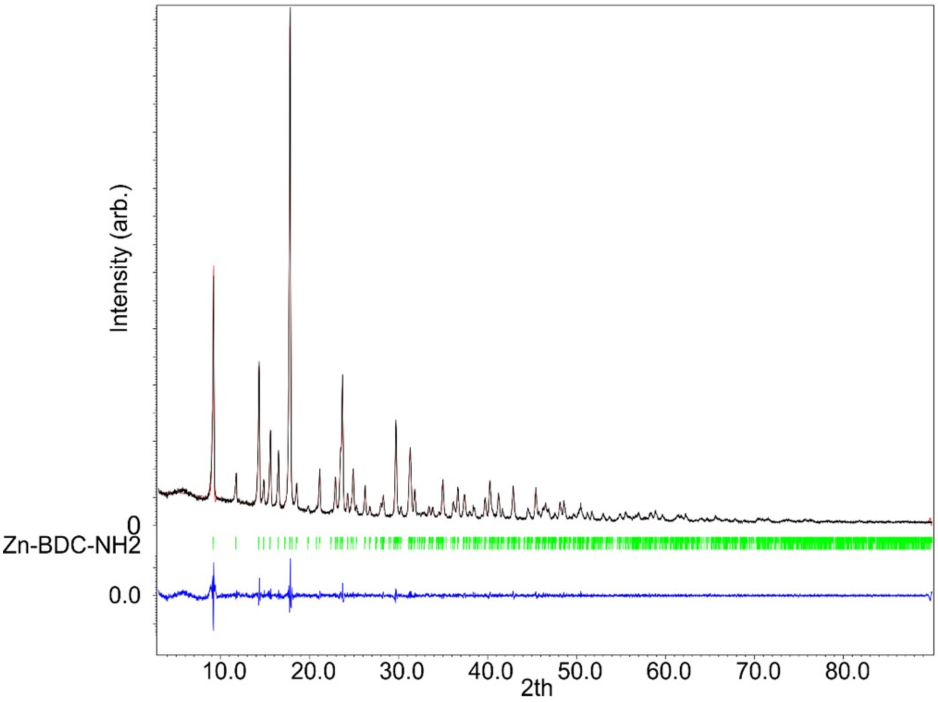

Figure S29. Le bail profile fitting for **TF-Silα@Zn-BDC-NH_2**. Black = observed data, red = calculated profile, green = allowed Miller indices, blue = Obs-Calc difference. Unit cell matches that of published **Zn-BDC-NH_2** CCDC 607821

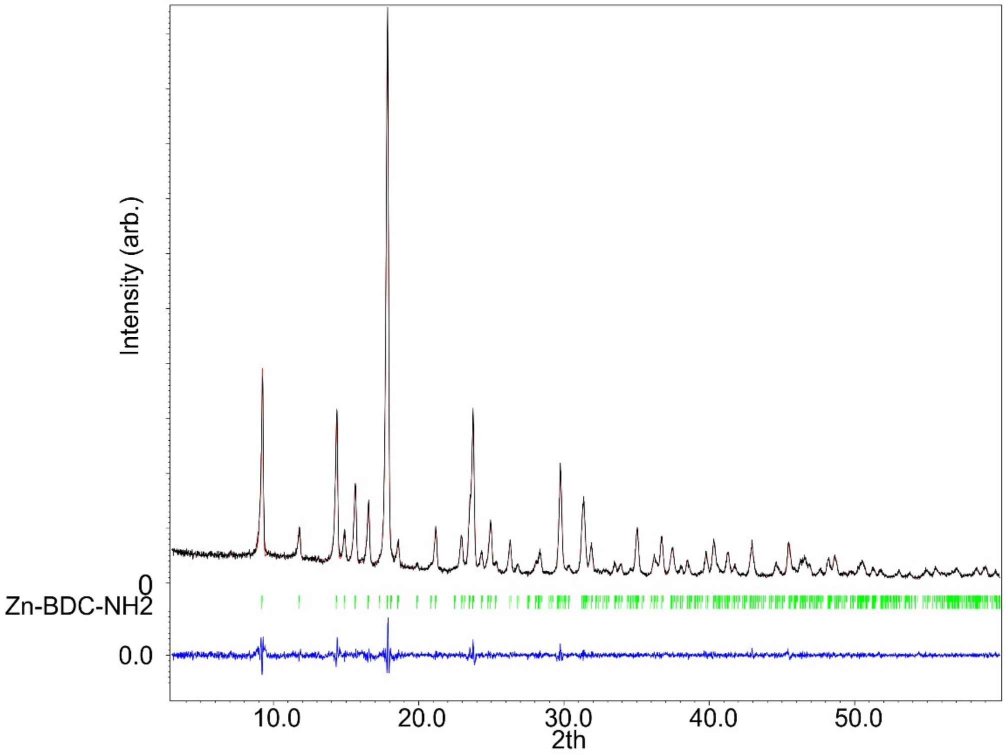

Figure S30. Le bail profile fitting for **Zn-BDC-NH$_2$**. Black = observed data, red = calculated profile, green = allowed Miller indices, blue = Obs-Calc difference. Unit cell matches that of published **Zn-BDC-NH$_2$** CCDC 607821

Table S6. Refined unit cell parameters

Sample	ZIF8	TF-Silα@ZIF-8	TF-Silα@Zn-BDC-NH$_2$	Zn-BDC-NH$_2$
a/Å	24.0976(10)	10.507(2)	9.5555(8)	9.5446(9)
b/Å	16.9750(9)	12.308(3)	6.4557(6)	6.4446(7)
c/Å	19.7171(8)	4.6617(7)	15.0242(12)	15.0007(14)
α/°	90	90	90	90
β/°	90	90	95.002(2)	95.002(3)
γ/°	90	90	90	90
Volume/Å^3	8065.4(6)	602.8(2)	923.27(13)	919.19(16)

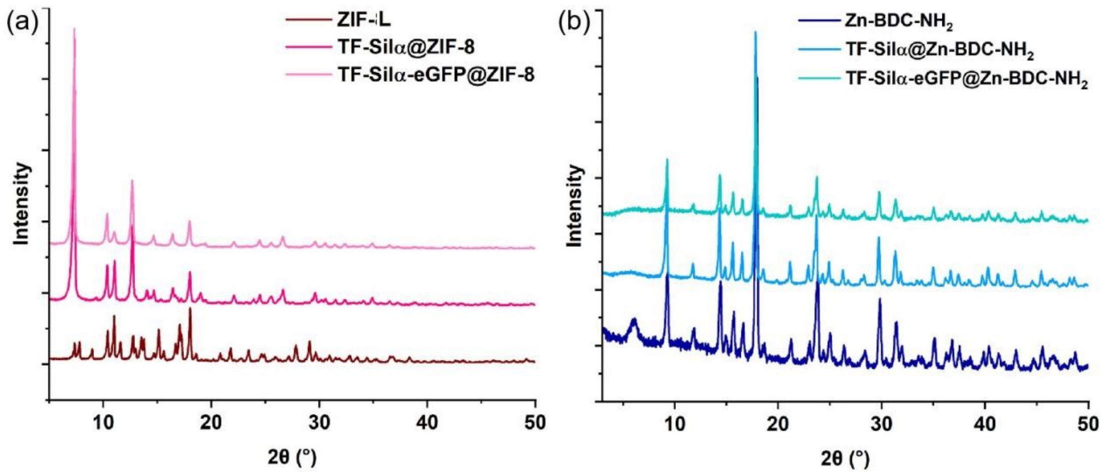

Figure S31. Stacked PXRD patterns of (a) **ZIF-L** (wine), **TF-Silα@ZIF-8** (pink) and **TF-Silα-eGFP@ZIF-8** (light pink); (b) **Zn-BDC-NH$_2$** (navy), **TF-Silα@Zn-BDC-NH$_2$** (blue) and **TF-Silα-eGFP@Zn-BDC-NH$_2$** (cyan).

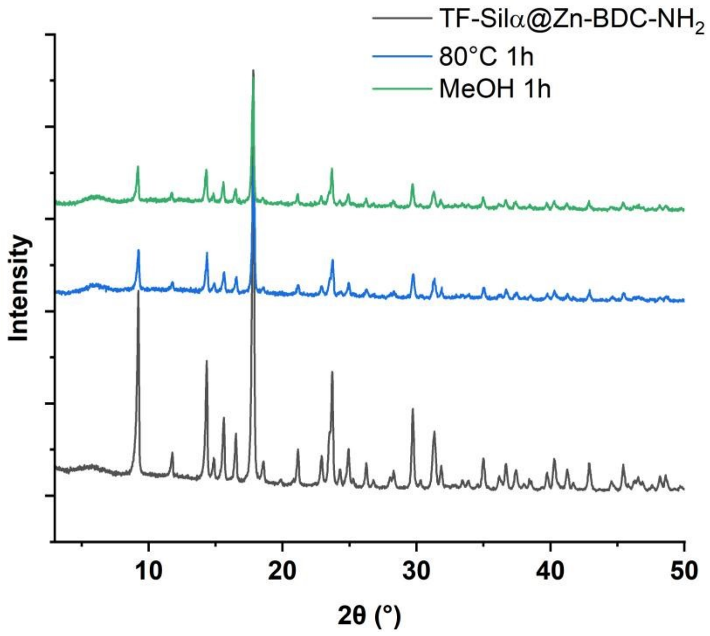

Figure S32. PXRD patterns of native **TF-Silα@Zn-BDC-NH$_2$** (black), **TF-Silα@Zn-BDC-NH$_2$** after exposure to high temperature (80°C, blue) or organic solvent (MeOH, green) for 1 hour. Both spectra confirm that the material remains crystalline following heat and organic solvent treatment, and there is no change to the crystal structure.

S4.7 Particle size analysis via Dynamic light scattering (DLS) and scanning electron microscopy (SEM)

For DLS measurements, the vacuum dried sample was ground and resuspended in water. The mixture was sonicated for 5 min to help suspension. For SEM, the dried sample was ground and resuspended in methanol before sonication for 5 min. The suspension was added to conductive carbon tape which was attached to aluminium stubs. The methanol was allowed to evaporate before coating the sample with platinum for SEM analysis.

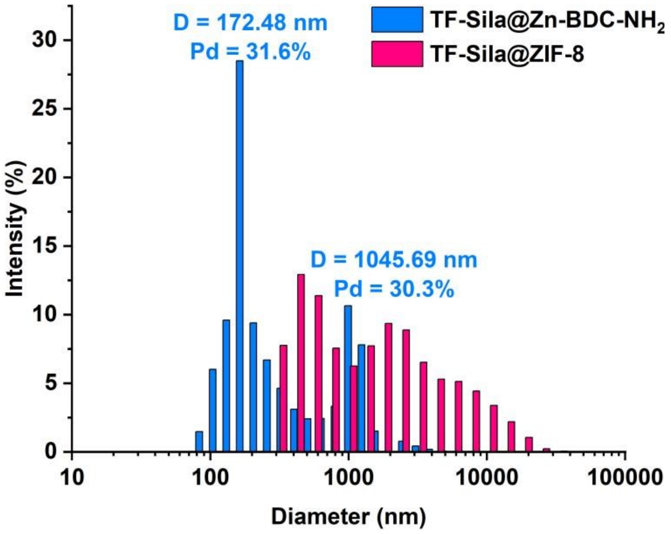

Figure S33. Particle size distribution of **TF-Silα@ZIF-8** and **TF-Silα@Zn-BDC-NH$_2$** measured with DLS. The **TF-Silα@ZIF-8** particles displayed an extremely broad polydispersity and were observed visually to precipitate from solution.

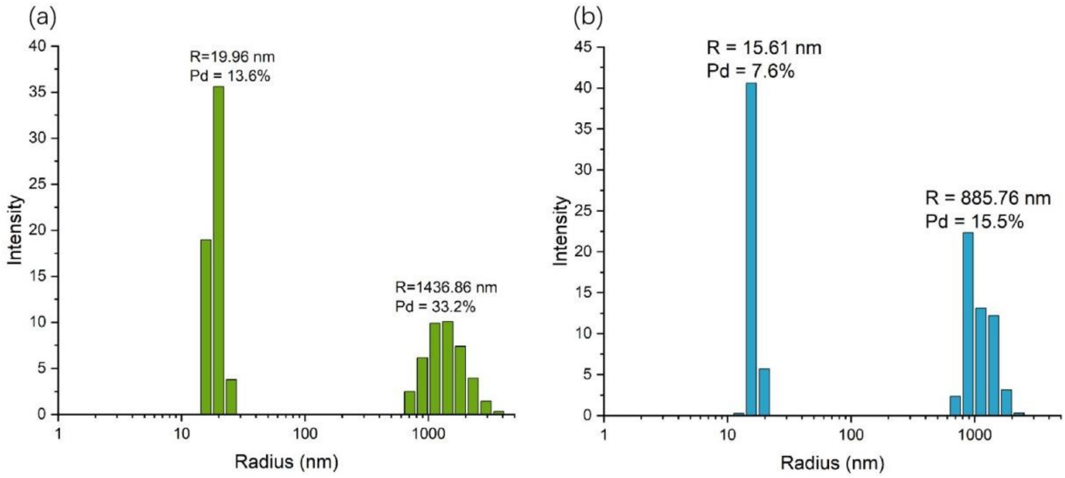

Figure S34. Size distribution of **TF-Silα@Zn-BDC-NH$_2$** particles with a protein loading content (LC%) of (a) LC = 10% (b) LC = 25%. Both experiments were carried out with particle concentration of 1mg.mL^{-1}. The distribution was averaged from 3 measurements.

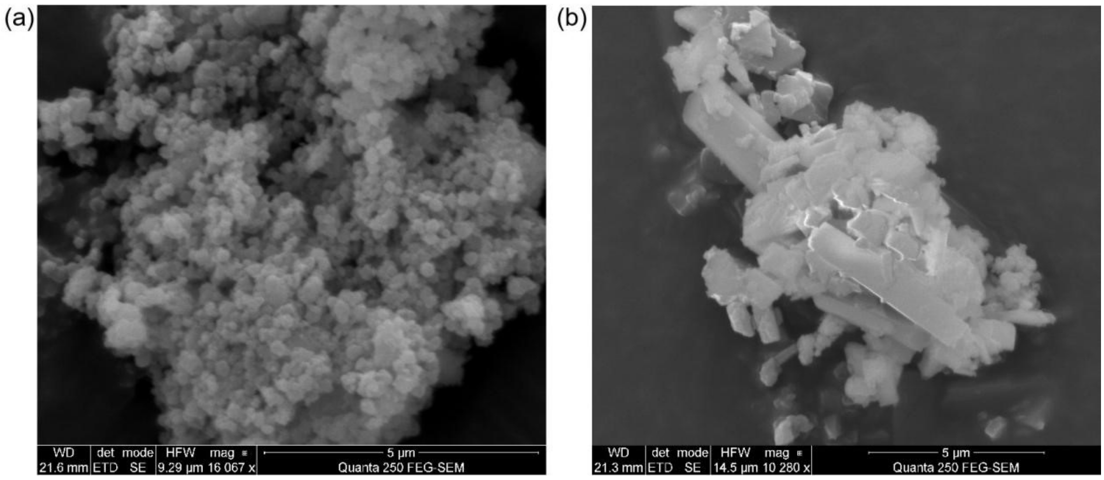

Figure S35. SEM images of (a) **TF-Silα@ZIF-8** and (b) **TF-Silα@ Zn-BDC-NH$_2$**. The **TF-Silα@ZIF-8** particles were observed as dodecahedron particles which clumped together to form larger aggregates. Conversely, the **TF-Silα@ Zn-BDC-NH$_2$** particles present as plates with an average diameter of 3 µm.

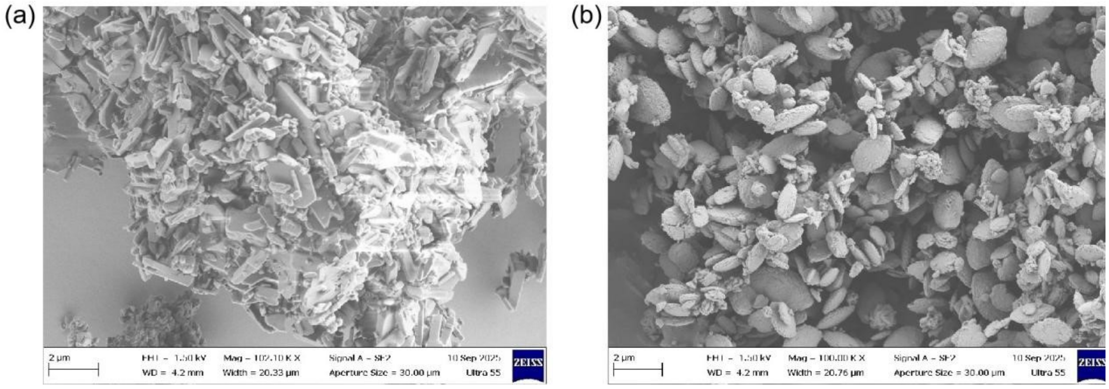

Figure S36. SEM images of (a) **Zn-BDC-NH$_2$** and (b) **ZIF-L**. **Zn-BDC-NH$_2$** displays plate-shaped particles similar as **TF-Silα@ Zn-BDC-NH$_2$**, whereas **ZIF-L** exhibits characteristic leaf-like morphology.

S5. Activity study of free and encapsulated TF-Silα

S5.1 Hydrolytic activity of TF-Silα

To measure the hydrolytic activity of free enzyme, 1 mM stock solution of TBDMS-OMeNp was prepared in 1,4-dioxane. The stock solution was diluted with dioxane to make a series of concentrations (50- 1000 µM) and each concentration (20 µL) was added to a microtiter plate and made up to 100 µL with Tris buffer. Then 100 µL enzyme solution (1 mg.mL^{-1}) or Tris buffer (negative control) was added to appropriate wells. Absorbance at 414 nm was measured by UV-Vis spectrophotometer every 10 min over 20 h at 22 °C. The plate was shaken continuously throughout the experiment. Each assay was performed in triplicate and the absorbance was averaged for each time point. The background hydrolysis from the negative control was subtracted

from the enzyme samples to give the net concentration of product that was attributable to enzyme-catalysed hydrolysis. 2-Methyl-4-nitrophenol was used for assay calibration.

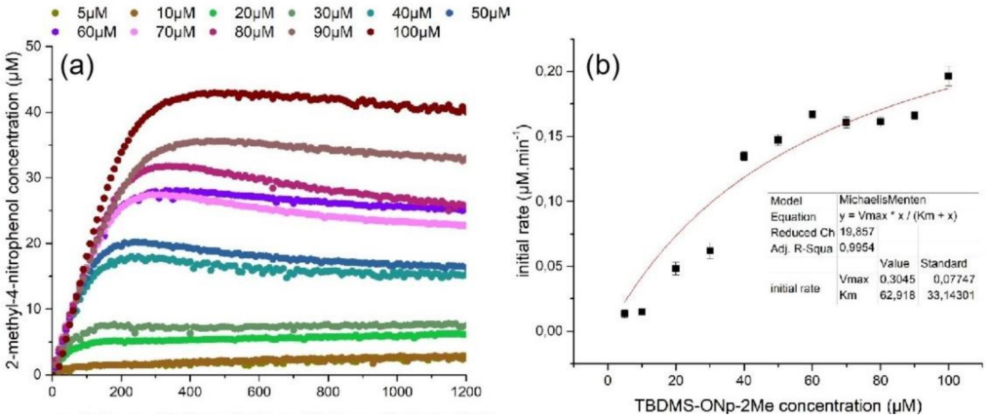

Figure S37. Concentration of (a) 2-methyl-4-nitrophenol and formed as a function of time using different concentrations of substrates and 67 µM of TF-Silα. The background hydrolysis from the negative control was subtracted from the enzyme samples. (b) Michaelis-Menten fitting for TBDMSONp-2Me. Error bars represent standard error of mean.

S5.2 Hydrolytic activity of TF-Silα@MOF composites

S.5.2.1 Colloidal Stability of TF-Silα@MOFs

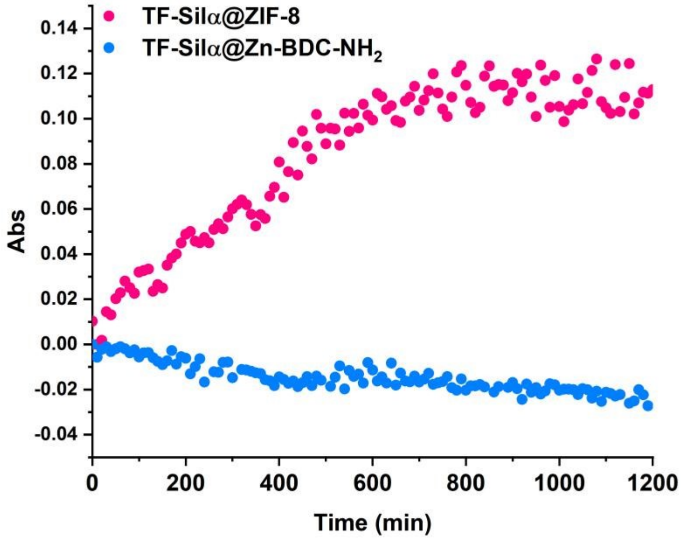

Figure S38. Colloid stability of **TF-Silα@ZIF-8** (pink) and **TF-Silα@Zn-BDC-NH$_2$** (blue). Both particles were re-suspended in Tris buffer (Table S1) to make a suspension containing 0.5 mg.mL^{-1} enzyme equivalent. The suspension was shaken at 22°C for 20h and absorbance at 414 nm was measured every 10 min. The initial absorbance at t=0 was subtracted from each data point. The results were averaged from 3 replicates.

The protocol was the same as described in S5.1, except that enzyme solution was replaced by **TF-Silα@MOF** suspension (containing 1 mg.mL^{-1} enzyme equivalent), which was prepared by re-suspending **TF-Silα@MOF** particles in Tris buffer (Table S1) and sonicating for 5 minutes. Background hydrolysis from the negative control and the absorbance of **TF-Silα@MOF** particles were both subtracted from the encapsulated enzyme samples. 2-Methyl-4-nitrophenol was used for assay calibration.

S.5.2.2 Hydrolytic Activity of TF-Silα@ZIF-8

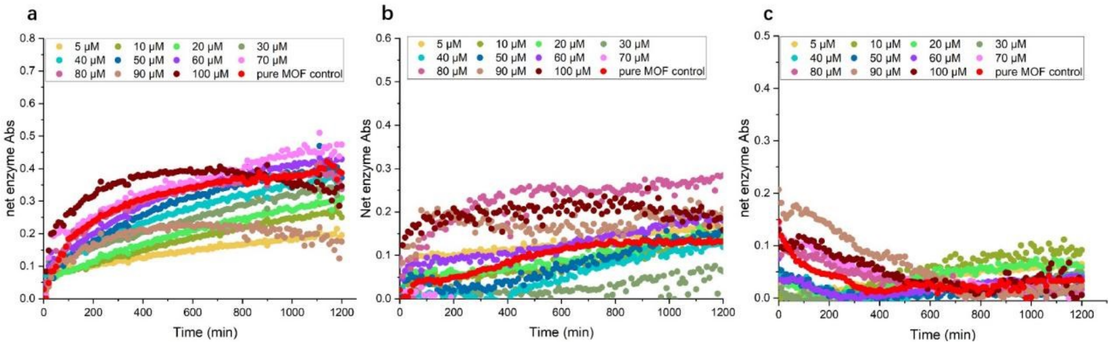

Figure S39. Time-coursed hydrolysis of TBDMS-OMeNp catalyzed by **TF-Silα@ZIF-8** (3.4 mg.mL^{-1}, containing 67 μM encapsulated TF-Silα). The background hydrolysis was subtracted from **TF-Silα@ZIF-8** group. Figure a, b, c represent three independant measurements.

In the hydrolysis catalyzed by enzyme@MOF partcles, we assume

$$A_{enzyme@MOF} = A_{background} + A_{MOF} + A_{net\ enzyme}$$

where $A_{enzyme@MOF}$ is the total absorbance in enzyme@MOF group, $A_{background}$ is background hydrolysis from negative control, A_{MOF} refers to the light scattering caused by MOF particles, and $A_{net\ enzyme}$ is the enzyme-catalyzed hydrolysis. As shown in Figure S34, background hydrolysis was subtracted from the **enzyme@MOF** absorbance. For all substrates concentrations, the absorbance curve resembles the curve of pure MOF control, and the final absorbance for different concentrations are quite similar. This suggests the enzyme encapsulated in **ZIF-8** might be deactivated ($A_{net\ enzyme}$ = 0), and the absorbance in each group is mainly caused by the MOF-induced light scattering.

S5.2.3 Hydrolytic activity of TF-Silα@Zn-BDC-NH_2

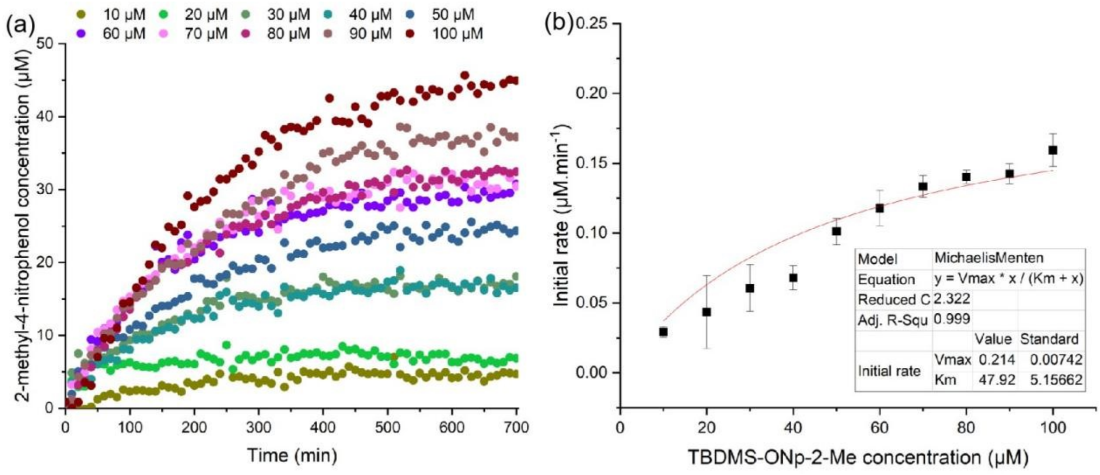

Figure S40. (a) One example of time-coursed hydrolysis of TBDMS-OMeNp catalyzed by **TF-Silα@Zn-BDC-NH_2** (4.9 mg.mL^{-1}, containing 67 μM encapsulated TF-Silα). The background hydrolysis and absorbance of **TF-Silα@Zn-BDC-NH_2** particles were subtracted. (b) One example of the Michaelis-Menten fitting. Error bars represent standard error of mean.

S5.3. Effectiveness factor of encapsulated TF-Silα

The degree of mass transfer to immobilized enzyme for enzyme kinetics was expressed in terms of effectiveness factor (Ƞ (%))

$$Effectiveness\ factor\ (\%) = \frac{V_{immobilized\ form}}{V_{free\ form}} \times 100$$

where, $V_{immobilized\ form}$ and $V_{free\ form}$ are the reaction rates catalyzed by enzymes in immobilized and free form.[19, 20]

Table S7. Reaction rate of TF-Silα ($V_{free\ form}$) and **TF-Silα@Zn-BDC-NH_2** ($V_{immobilized\ form}$) during first 100 minutes and effectiveness factor of **TF-Silα@Zn-BDC-NH_2**.

Substrate concentration (μM)	$V_{free\ form}$ (μM.min^{-1})	$V_{immobilized\ form}$ (μM.min^{-1})	Ƞ (%)
20	0.04819±0.00484	0.0276±0.00517	57.27329±10.72837
30	0.0619±0.00618	0.0422±0.00561	68.17447±9.06300
40	0.1347±0.00347	0.076533±0.00572	56.81762±4.24647
50	0.14728±0.00414	0.07985±0.0087	54.21646±5.90712
60	0.16689±0.00284	0.10124±0.00732	60.66271±4.38612

S5.4 Condensation activity of TF-Silα and TF-Silα@MOF composites

TF-Silα solution and PBS buffer were lyophilized as described previously.[2] A substrate stock was prepared by mixing 3-methoxyphenol (1.26 mmol) and triethylsilanol (6.33 mmol) in 3 mL of octane. 100 µL of this aliquot was then added to a glass vial containing lyophilized enzyme (0.5 mg) or **TF-Silα@MOF** with equivalent enzyme amount. Lyophilized buffer and pure MOF were used as their corresponding negative controls. The vials were crimp sealed and heated at 95 °C for 72 hrs while shaking at 650 rpm. Then the reaction mixture was diluted with 1 mL of hexane and centrifuged (17,000× g, 10 min) before analysis of the supernatant by GC-MS. All the reactions were performed in triplicate and GC-MS was calibrated with synthesized standards to quantify the conversion rates.

S5.5. GC-MS calibration

The synthesized calibrants were dissolved in *n*-hexane to prepare a 40 mM stock, which was further diluted with hexane to a series of working solutions. Each solution was injected into the GC-MS, and the peak area from the chromatogram was plotted against the concentration. The resulting calibration curve was used to determine the unknown product concentration.

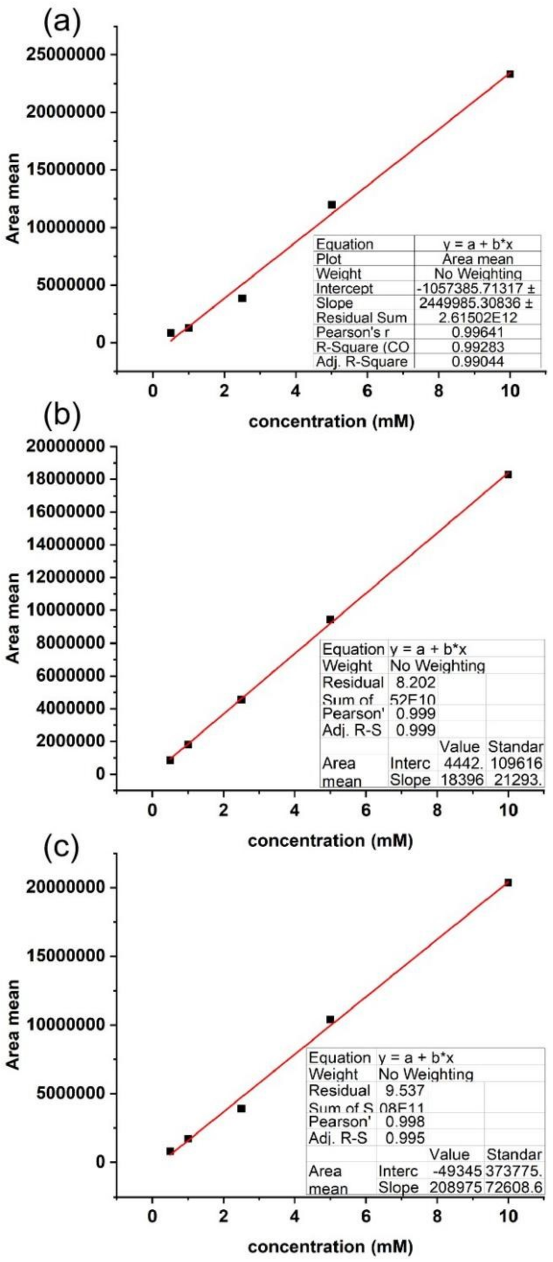

Figure S41. One example of the GC-MS calibration for (a) triethyl(phenoxy)silane (b) triethyl(3-methoxyphenoxy)silane and (c)triethyl(4-methoxyphenoxy)silane. Peak area was dependant on the instrument status, thus fresh calibration curves were prepared each day. Samples and calibrants were measured with the same method profile.

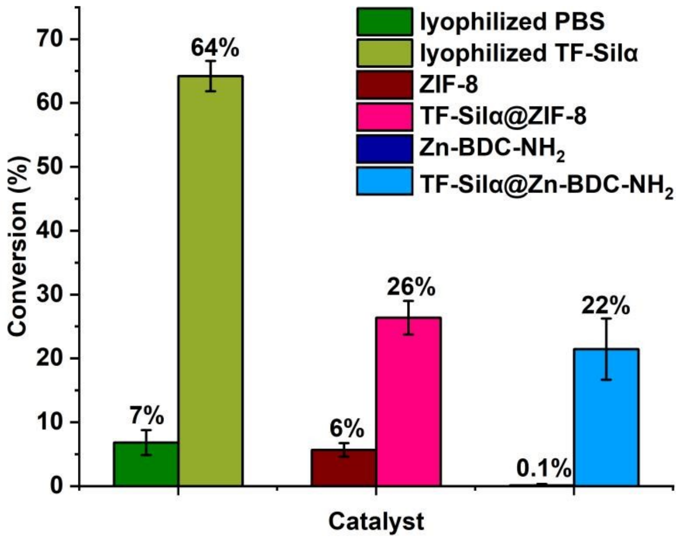

Figure S42. Percentage conversions of 3-methoxyphenol to triethyl(m-methoxyphenoxy)silane. The reaction was run in the presence of lyophilized TF-Silα, **TF-Silα@ZIF-8** and **TF-Silα@Zn-BDC-NH$_2$** with 10 % LC. Negative controls (lyophilized buffer and pure MOFs) were also acquired.

S5.6 Comparison of stability of free and biomineralised TF-Silα

S5.6.1 Thermal stability:

TF-Silα@Zn-BDC-NH$_2$ was resuspended in Tris buffer and sonicated for 5 minutes to make a suspension containing 1 mg.mL^{-1} enzyme. This suspension or the TF-Silα solution (1 mg.mL^{-1} in Tris buffer, Table S1) were heated at 50°C or 80°C for 1 hour while shaking at 650 rpm. After heat treatement 100 µL of enzyme solution or **enzyme@MOF** suspension was added to the microtitre plate, and their hydrolytic activity was measured with 100 µM TBDMS-OMeNp. The activity was calculated as the initial rate during the first 100 minutes, and the non-treated control group was normalized to 100% activity.

S5.6.2 Chemical stability:

0.5 mg lyophilized enzyme (see S2) and **TF-Silα@Zn-BDC-NH$_2$** with an equivalent amount of enzyme were individually suspended in 1 mL of organic solvents (dioxane, methanol, tetrahydrofuran) and shaken at 650 rpm for 2 hours. The solvents were then evaporated under vacuum, and the enzyme or **enzyme@MOF** were resuspended in 0.5 mL Tris buffer. 100 µL of this enzyme solution or **enzyme@MOF** suspension were then used to catalyze the hydrolysis of 100 µM TBDMS-OMeNp. The activity was calculated in the same manner as above.

S5.6.3 Long-term stability:

TF-Silα solution (1 mg.mL^{-1} in Tris buffer) and a **TF-Silα@Zn-BDC-NH$_2$** suspension (containing 1 mg.mL^{-1} enzyme) were stored at room temperature for 16 - 27 days before measuring their hydrolysis of 100 µM TBDMS-OMeNp. The activity was calculated in the same manner as above.

S5.7 Determination of half-life for free and encapsulated TF-Silα

The deactivation of enzyme was approximated to a non-reversible, first order process, whereby:
$A_{(t)} = A_0 e^{(-kt)}$
Where $A_{(t)}$ is activity at time t, and A_0 is initial activity at day 0. Therefore, by plotting $Ln(A(_{t})/A_0))$ against time t, k can be determined from the negative slope. Since half-life ($t_{1/2}$) refers to the time required to reach half initial activity, $t_{1/2}$ can be calculated by ln2/*k*. Thus the half-life of free enzyme was determined to be (7.3 ± 0.4) days, while **TF-Silα@Zn-BDC-NH$_2$** has a half-life of (32.2 ± 5.8) days at room temperature.

S5.8 Reusability test of free and biomineralised TF-Silα

TF-Silα@Zn-BDC-NH$_2$ was resuspended in Tris buffer (Table S1) and sonicated for 5 min to make a suspension containing 1 $mg.mL^{-1}$ enzyme. To quantify enzyme activity, 100 µL of this suspension was added to a 96 multiwell plate containing 80 µL Tris buffer, followed by 20 µL TBDMS-OMeNp dioxane solution to make final substrate concentration of 100 µM. The activity was calculated as the initial rate during the first 100 minutes with the first cycle normalized to 100% activity.
After a cycle of the assay (20 hours), the plate was centrifuged (4000 rpm, 10 min) and the supernatant was discarded. The **enzyme@MOF** precipitate was washed twice with Tris buffer (200 µL x 2) by repeating suspension and centrifugation. Then **TF-Silα@Zn-BDC-NH$_2$** was resuspended in Tris buffer for the next cycle of catalysis.
For free enzyme, a TF-Silα solution (1 $mg.mL^{-1}$ in Tris buffer) was shaken at 22 °C for different time periods (0 hours, 20 hours, 40 hours, 60 hours, 80 hours) prior to commencement of the hydrolysis assay, thus free enzyme solution was exposed to room temperature for the equivalent amount of time as the recycled **TF-Silα@Zn-BDC-NH$_2$**. The enzyme activity was then assayed in the same manner as above with TBDMS-OMeNp.

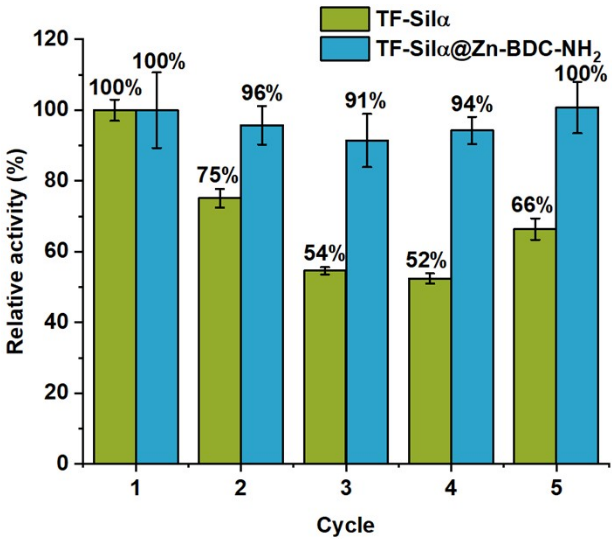

Figure S43. Hydrolytic activity of recycled TF-Silα and **TF-Silα@Zn-BDC-NH$_2$**. The results are averaged from three measurements, the error bars represent standard error of the mean.

S5.9 Optimization of condensation reaction

To select the optimum conditions for the condensation reaction, different solvents, substrates, enzyme treatments, reaction time and temperature were screened as described below.

S5.9.1 Solvent screening:

Phenol (1.26 mmol) and triethylsilanol (6.33 mmol) were dissolved in 3 mL of toluene or octane to make substrate stock solutions. 100 µL of each solution was added to lyophilized enzyme (0.5 mg) or buffer and heated at 75 °C for 72 hours while shaking at 650 rpm.

S5.9.2 Enzyme storage effect screening:

Freshly purified TF-Silα and frozen enzyme (enzyme solution stored at -80 °C) were separately lyophilized as described before. In addition, some previously lyophilized TF-Silα (lyophilized then stored in freezer) was also used and the activity of the three preparations was compared. Phenol (1.26 mmol) and triethylsilanol (6.33 mmol) were dissolved in 3 mL of octane to make substrate stock. Then 100 µL of this aliquot was added to the lyophilized enzyme preparations (containing 0.5 mg TF-Silα) or lyophilized buffer without enzyme and heated at 75 °C for 72h while shaking at 650 rpm. The condensation product was then quantified by GC-MS as above.

S5.9.3 Reaction time and temperature screening:

A substrate stock was prepared by mixing phenol (1.26 mmol) and triethylsilanol (6.33 mmol) in 3 mL of octane. 100 µL of this aliquot was then added to lyophilized enzyme (containing 0.5 mg TF-Silα) or TF-Silα@MOF with an equivalent enzyme amount. Lyophilized buffer and MOF without enzyme were used as their corresponding negative controls. Each material was then shaken (650 rpm) at 75 °C for 3 days, 75 °C for 6 days, and 95 °C for 3 days, respectively, to select the best reaction time and temperature according to the highest conversion.

S5.9.4 Substrates screening:

6.33 mmol triethylsilanol and 1.26 mmol phenol derivatives (phenol, 3-methoxyphenol, 4-methoxyphenol) were dissolved in 3 mL of octane to make substrate stock solutions. 100 µL of each solution was then added to lyophilized enzyme (0.5 mg) or buffer and heated at 95 °C for 72 hours while shaking at 650 rpm.

All the reactions were performed in triplicate and analyzed with GC-MS as described above.

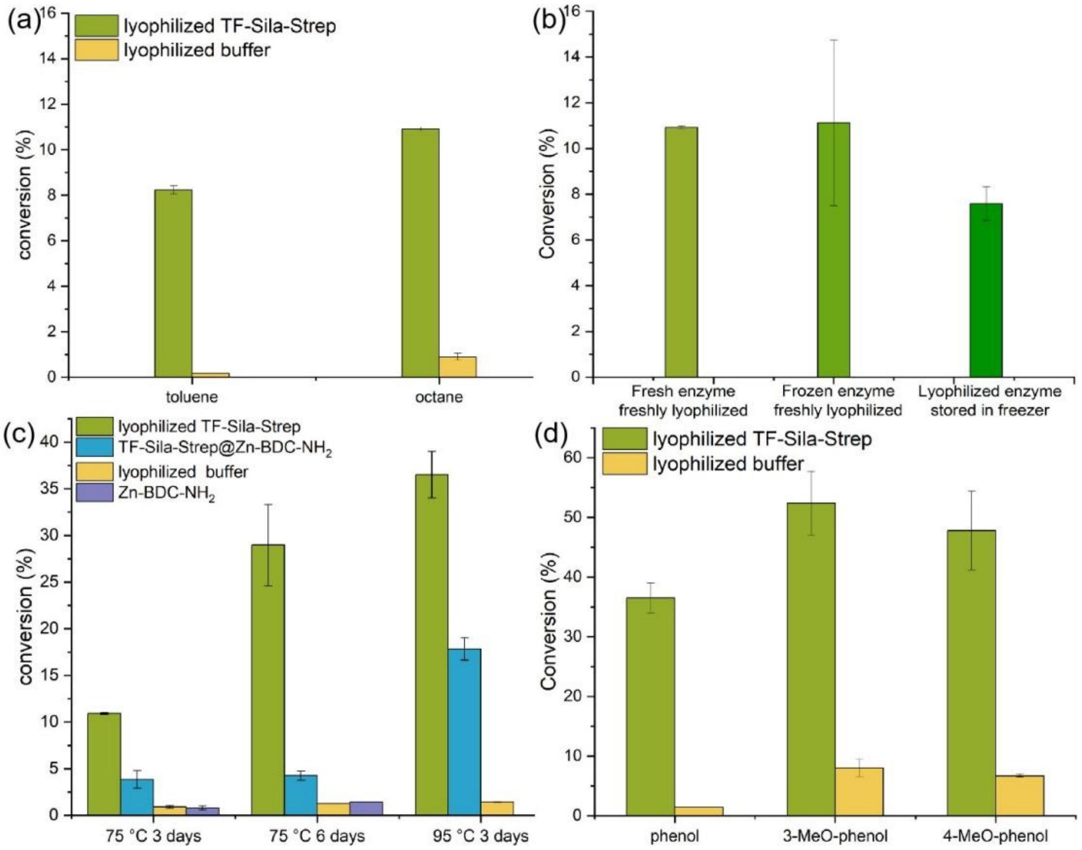

Figure S44. Influence of different: (a) solvents; (b) enzyme treatments; (c) temperature and reaction time; and (d) substrate on percentage conversion. Each assay was performed in triplicate and error bars represent the standard deviation.

S6. References:

1. E. I. Sparkes, R. A. Kettles, C. S. Egedeuzu, N. L. Stephenson, S. A. Caslin, S. Y. Tabatabaei Dakhili and L. S. Wong, *Biomolecules*, 2020, **10**, 1209.
2. E. I. Sparkes, C. S. Egedeuzu, B. Lias, R. Sung, S. A. Caslin, S. Y. Tabatabaei Dakhili, P. G. Taylor, P. Quayle and L. S. Wong, *Catalysts*, 2021, **11**, 879.
3. Y. Lu, C. S. Egedeuzu, P. G. Taylor and L. S. Wong, *Biomolecules*, 2024, **14**, 492.
4. Y.-B. Miao, Q. Zhong and H.-X. Ren, *Anal. Bioanal. Chem.*, 2022, **414**, 8331-8339.
5. A. Barth, *BBA - Bioenergetics*, 2007, **1767**, 1073-1101.
6. D. Tocco, D. Chelazzi, R. Mastrangelo, A. Casini, A. Salis, E. Fratini and P. Baglioni, *J. Colloid Interface Sci.*, 2023, **641**, 685-694.
7. S. Y. Tabatabaei Dakhili, S. A. Caslin, A. S. Faponle, P. Quayle, S. P. de Visser and L. S. Wong, *Proc. Natl Acad. Sci.*, 2017, **114**, E5285-E5291.

8. F. Lyu, Y. Zhang, R. N. Zare, J. Ge and Z. Liu, *Nano Lett*, 2014, **14**, 5761-5765.
9. A. B. Elmas Kimyonok and M. Ulutürk, *J. Energ. Mater.*, 2016, **34**, 113-122.
10. Y. Wang, S. Tadepalli, H. Baldi, J. Morrissey and S. Singamaneni, *Mater. Interfaces,* **2024**, *1*, 47–57
11. Rigaku Oxford Diffraction, 2025.
12. G. M. Sheldrick, *Acta Cryst. A71*, 2015, 3-8.
13. L. J. Bourhis, O. V. Dolomanov, R. J. Gildea, J. A. Howard and H. Puschmann, *Acta Cryst. A*, 2015, **71**, 59-75.
14. O. V. Dolomanov, L. J. Bourhis, R. J. Gildea, J. A. K. Howard and H. Puschmann, *J. App. Cryst.*, 2009, **42**, 339-341.
15. A. Saha, S. S. Nia and J. A. Rodríguez, *Chem. Rev.* , 2022, **122**, 13883.
16. X. Wang, S. P. Singh, T. Zhang, R. Andrews, M. G. Lizio and I. A. Riddell, *Inorg. Chem.*, 2024, **63**, 9801-9808.
17. A. A. Coelho, *J. Appl. Cryst.*, 2017, **50**, 1323-1330.
18. V. Petříček, L. Palatinus, J. Plášil and M. Dušek, *Z. Kristallogr. Cryst. Mater.*, 2023, **238**, 271-282.
19. A. B. Muley, A. S. Thorat, R. S. Singhal and K. Harinath Babu, *Int. J. Biol. Macromol.*, 2018, **118**, 1781-1795.
20. A. B. Muley, K. H. Mulchandani and R. S. Singhal, in *Methods in Enzymology*, Elsevier, 2020, pp. 39-79.
